# APOE4 promotes cerebrovascular fibrosis and amyloid deposition via a pericyte-to-myofibroblast transition

**DOI:** 10.1101/2025.09.04.674192

**Published:** 2025-09-09

**Authors:** Braxton R. Schuldt, Dominic Haworth-Staines, Andrea Perez-Arevalo, Diede W.M. Broekaart, Leon Wang, Georgia Gallagher, Grace Rabinowitz, Alison M. Goate, Towfique Raj, Ana C. Pereira, Joel W. Blanchard

**Affiliations:** 1 Nash Family Department of Neuroscience and Friedman Brain Institute, Icahn School of Medicine at Mount Sinai, New York, NY, 10029, USA.; 2 Ronald M. Loeb Center for Alzheimer’s Disease, Icahn School of Medicine at Mount Sinai, New York, NY, 10029, USA.; 3 Institute for Regenerative Medicine, Black Family Stem Cell Institute, Icahn School of Medicine at Mount Sinai, New York, NY, 10029, USA; 4 Department of Neurology, Icahn School of Medicine at Mount Sinai, New York, NY, 10029, USA

**Keywords:** APOE4, cerebrovascular degeneration, pericyte, myofibroblast, TGF-β, fibrosis, fibronectin, cerebral amyloid angiopathy, Alzheimer’s disease, iPSC modeling

## Abstract

Cerebrovascular disease is a major but poorly understood feature of Alzheimer’s disease (AD). The strongest genetic AD risk factor, APOE4, is associated with cerebrovascular degeneration, including vascular amyloid deposition and fibrosis. To uncover how APOE4 promotes cerebrovascular pathology, we generated a single-cell transcriptomic atlas of human brain vasculature. In APOE4 carriers, pericyte abundance was significantly reduced and accompanied by the emergence of a myofibroblast-like cell population co-expressing contraction and extracellular matrix genes. Immunostaining confirmed non-vascular myofibroblasts in APOE4 human and mouse brains. We show that APOE4 pericytes transition into myofibroblasts that secrete fibronectin, which promotes vascular amyloid accumulation. Computational and experimental analyses identified elevated TGF-β signaling as the driver of this pericyte-to-myofibroblast transition. Inhibition of TGF-β restored pericyte coverage and reduced vascular fibrosis and amyloid to APOE3 levels, revealing a targetable mechanism linking APOE4 to cerebrovascular pathology in AD.

## Summary

Cerebrovascular disease is a prominent but poorly understood component of Alzheimer’s disease (AD). The strongest genetic risk factor for AD, APOE4, is associated with multiple cerebrovascular pathologies, including vascular amyloid accumulation and fibrosis. APOE4 also renders the cerebrovasculature fragile to the point where the only disease-modifying AD therapeutic, anti-amyloid monoclonal antibodies, are warned against use in APOE4 carriers due to a high risk of hemorrhage and edema. To determine how APOE4 impacts cerebrovascular cells and pathogenesis, we generated a high-resolution single-cell transcriptomics atlas of human cerebrovasculature from APOE4 carriers and non-carriers. In APOE4 individuals, we found that the number of microvascular pericytes was significantly reduced. Notably, loss of pericytes in APOE4 carriers was accompanied by the emergence of a unique population of mural cells characterized by high expression of contraction and extracellular matrix (ECM) genes, hallmarks of myofibroblasts. Immunostaining revealed the presence of myofibroblasts surrounding the microvasculature in APOE4 human hippocampi that were absent in age-matched APOE3/3 controls, even in cases of AD. Myofibroblast presence coincided with a significant increase in fibronectin and amyloid surrounding the vasculature. Myofibroblasts were also present around the microvasculature of aged APOE4/4 but not APOE3/3 mice, suggesting myofibroblast appearance is a direct effect of the APOE4 genotype and not a technical artifact of processing human tissue. To determine the mechanisms and functional implications of APOE4-mediated myofibroblast, we leveraged a vascularized human brain tissue (miBrain) derived from induced pluripotent stem cells. Similar to the post-mortem human brain, APOE4/4 miBrains showed significantly reduced microvascular pericyte coverage, coinciding with the emergence of myofibroblasts co-expressing ECM and contractile genes. Lineage tracing and genetic mixing experiments confirmed that the myofibroblasts emerge from APOE4 pericytes and secrete fibronectin-rich ECM. Knocking down fibronectin in APOE4 mural cells significantly reduced vascular amyloid accumulation. To determine how myofibroblast-like cells arise in the APOE4 brain, we performed computational analysis (NicheNet) on our post-mortem human single-cell transcriptomics cerebrovascular atlas. This predicted that TGF-β is the top causal driver of the pericyte-to-myofibroblast transition. Consistent with this prediction, we found that TGF-β ligands in astrocytes and receptors in mural cells are significantly upregulated compared to APOE3 controls. Chemical and genetic inhibition of TGF-β signaling in APOE4 miBrains significantly reduced myofibroblast presence, while concurrently increasing pericyte microvascular coverage and ultimately lowering vascular fibrosis and amyloid accumulation to APOE3 levels. Using a comprehensive three-pronged approach incorporating analysis of human post-mortem brain, APOE humanized mice, and human iPSC-derived vascularized brain tissue, we demonstrate that APOE4 in mouse and human brain tissue causes increased TGF-β signaling, promoting a pericyte-to-myofibroblast transition that leads to vascular fibrosis and amyloid deposition. This provides critical insight into the mechanisms underlying APOE4 cerebrovascular dysfunction, highlighting new diagnostic and therapeutic strategies for a major AD risk population.

## Introduction

Alzheimer’s disease (AD) is a common and devastating neurodegenerative disorder characterized by progressive cognitive decline and brain atrophy. The vast majority of cases are classified as late-onset AD, where aging and genetic factors play a major role in disease risk^1^. Among these, the APOE4 allele stands out as the strongest genetic risk factor for AD identified through genome-wide association studies (GWAS) ^2^. Remarkably, APOE4 differs from the more common APOE3 allele by a single amino acid (Cys112→Arg112), yet individuals carrying two copies of APOE4 face up to 16-fold increased risk of developing AD^3^. Furthermore, 40–65% of all individuals with AD carry at least one APOE4 copy, underscoring its relevance to disease pathogenesis^4^. Understanding how APOE4 contributes to AD progression is therefore critical to developing targeted therapies for this large and vulnerable patient population.

Since its identification as an AD risk variant, APOE4 has been implicated in dysfunction across nearly all major brain cell types. In neurons, APOE4 drives tau hyperphosphorylation and death^5–8^, in microglia it promotes lipid accumulation and dysfunction^9–11^, in astrocytes it drives proinflammatory signalling^12–15^, and in oligodendrocytes it disrupts myelination via cholesterol dysregulation^16^. Despite these widespread effects, APOE4 may exert its most profound influence on the cerebrovasculature^17–21^. Perivascular amyloid accumulation, blood-brain-barrier (BBB) breakdown, and microvessel degeneration are among the earliest changes that occur during AD pathogenesis and directly contribute to disease progression^22–24^. Furthermore, these vascular defects directly impair brain homeostasis and accelerate cognitive decline^25–33^. Even in the absence of AD pathology, APOE4 carriers show increased cerebrovascular dysfunction, suggesting that the vascular effects of APOE4 may be an upstream driver of AD pathogenesis^25,34–37^. While the advent of anti-amyloid monoclonal antibodies marked a major therapeutic milestone, their use in APOE4 carriers is limited by the elevated risk of amyloid-related imaging abnormalities (ARIA), including cerebral hemorrhage and edema^38,39^. The vulnerability of APOE4 carriers to the only FDA-approved AD therapeutic underscores a critical need: preventing or reversing APOE4-associated cerebrovascular damage may be essential to slowing or halting AD progression in this high-risk population.

Cerebral blood vessels are composed of endothelial cells, mural cells, and a basement membrane, with additional structural and functional support from astrocytic endfeet. Each of these components has been independently implicated in APOE4-mediated cerebrovascular degeneration. In endothelial cells, APOE4 promotes inflammatory and prothrombotic signaling while impairing tight junctions and compromising BBB integrity^40,41^. Pericytes are key contributors to cerebral amyloid angiopathy (CAA) and BBB breakdown, with APOE4 expression in pericytes directly driving these pathologies^25,30,42–47^. Beyond these canonical vascular cell types, single-cell studies have revealed an unexpected diversity of fibroblasts within brain barrier structures, suggesting emerging roles in cerebrovascular homeostasis^48,49^. Supporting this, recent studies have identified protective genetic variants in vascular basement membrane components, such as fibronectin, that reduce AD risk in APOE4 carriers^50,51^. Moreover, the profibrotic cytokine TGF-β has been linked to vascular degeneration, further implicating dysregulation of the extracellular matrix in APOE4-associated cerebrovascular pathology^52–54^. Despite these findings across cell types and compartments of the APOE4 cerebrovasculature, the precise mechanisms through which APOE4 expression in vascular cells leads to cerebrovascular dysfunction and AD pathogenesis remain poorly defined.

To address this gap, we constructed a high-resolution transcriptomics atlas of the human cerebrovasculature by integrating previously published single-nuclei RNA-sequencing (snRNA-seq) datasets from APOE4 carriers and non-carriers. We complement this computational approach with postmortem immunostainings, isogenic iPSC models of the human brain, and humanized APOE knock-in mice. Through this comprehensive three-pronged approach (human postmortem, iPSC models, and APOE-TR mice), we discover that APOE4 promotes a pericyte-to-myofibroblast transition in the brain. We establish that elevated TGF-β signaling in the APOE4 brain drives this transition, resulting in a loss of pericyte coverage and excessive fibronectin deposition along the vasculature. We show that fibronectin secretion from APOE4 myofibroblasts directly promotes perivascular amyloid accumulation and correlates with an earlier age of AD diagnosis and more severe cerebral pathology. Importantly, we demonstrate that TGF-β inhibition in APOE4 human brain tissue reverses the pericyte-to-myofibroblast transition and reduces cerebrovascular fibrosis and amyloid accumulation, thus providing a direct mechanistic link between APOE4, TGF-β, vascular fibrosis, and AD pathogenesis.

## Results

### Harmonized single-nucleus atlas of the human APOE4 cerebrovasculature

**Figure 1.**
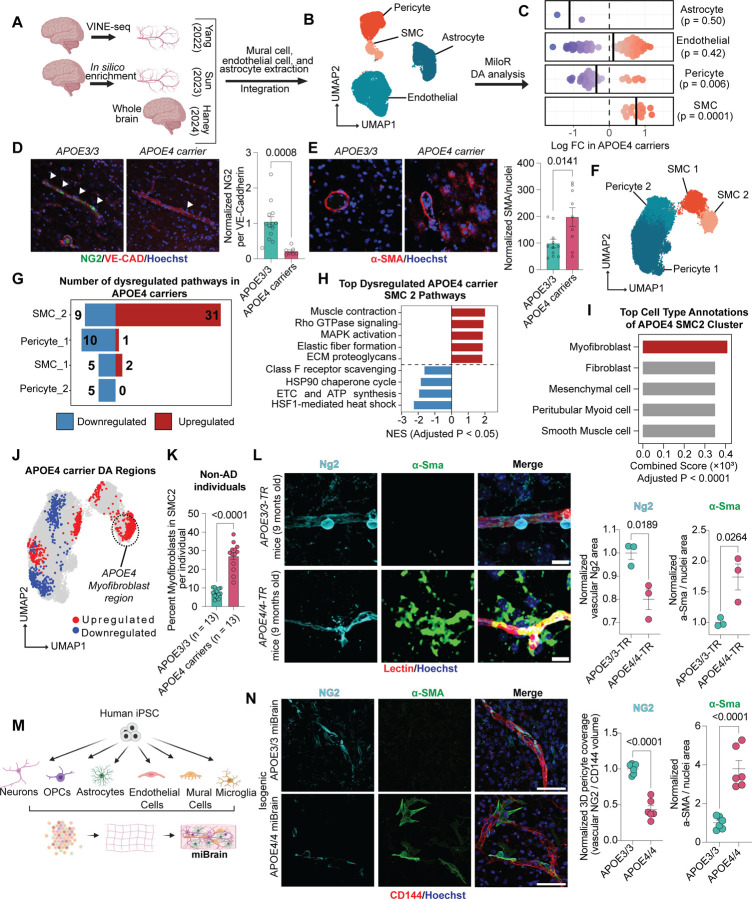
A myofibroblast-like mural cell population is enriched in the APOE4 brain. **A.** Schematic of cerebrovascular atlas generation from single nuclei RNA-sequencing of the post-mortem human brain in APOE4 carriers and non-carriers. **B.** UMAP of harmonized blood-brain barrier cell types. **C.** Significant differentially abundant (DA) cell neighborhoods per cell type in APOE4 carriers vs. non-carriers using the miloR package (spatial FDR < 0.05, positive logFC indicates enrichment in APOE4 carriers). Data points are individual cell neighborhoods. Solid vertical lines represent mean logFC for each cell type. A one-sample Wilcoxon signed-rank test with the Benjamini-Hochberg correction was performed on the distribution of logFC values for each cell type to test whether the median logFC significantly differed from 0. **D.** Representative images of post-mortem human hippocampus stained for markers of pericytes (NG2) and blood vessels (VE-CAD) in APOE3/3 and APOE4 carriers. Pericyte coverage for each genotype was calculated by quantifying the overlapping area of pericyte and blood vessel staining, expressed relative to APOE3/3. Data points represent individuals (N = 13 APOE3/3 and N = 7 APOE4 carriers). Bars are group means ± SEM. P-values were calculated using an unpaired two-tailed Student’s t-test. **E.** Representative images of APOE4 carrier and non-carrier post-mortem human hippocampus stained for the smooth muscle cell marker αSMA. The area positive for αSMA was quantified, normalized to nuclei, and expressed relative to APOE3/3. Data points represent individuals (N = 11 APOE3/3 and N = 9 APOE4 carriers). Bars are group means ± SEM. P values were calculated using an unpaired two-tailed Student’s t-test. **F.** UMAP of mural cell types after subclustering. **G.** Differential gene expression between APOE4 carriers and non-carriers for each mural cell type was performed using the MAST package. Genes were then ranked according to sign(logFC) * −log10(P-value) and input into the fgsea package using the REACTOME database. The number of significant pathways for each cell type was then calculated (FDR < 0.05). **H.** The top significantly upregulated and downregulated pathways in APOE4 carrier SMC_2 cells compared to APOE3/3 (FDR < 0.05, ranked by normalized enrichment score [NES]). Redundant pathways were merged to enhance readability. **I.** Top cell type annotations of SMC_2 cells from APOE4 carriers. The differentially expressed genes (p < 0.05, logFC > 0.25) upregulated in APOE4 SMC_2 cells from the pseudobulk approach in Extended Data 1G were input into EnrichR with the CellMarker database. Redundant cell types were merged to enhance readability. **J.** Differential abundance analysis on the mural cell clusters was performed with DAseq. Red and blue indicate enrichment and depletion in APOE4 carriers, respectively. Given the overlap of the APOE4-enriched SMC_2 region with ECM and contraction co-signatures, and the analyses performed in Extended Data 2B–D, this region was annotated as myofibroblasts. **K.** Subject-level predicted proportion of myofibroblasts in non-AD individuals, as estimated by the binomial generalized linear model described in Extended Data 2E. Data points represent individuals (N = 13 per genotype). Bars are group means ± SEM. P-values are derived from the binomial GLM. **L.** Representative images of small vessels (<6 μm) in the corpus callosum of age- (9 to 10 months of age) and sex-matched APOE3/3 and APOE4/4-TR mice stained for markers of blood vessels (lectin), myofibroblasts (αSma), and pericytes (Ng2). Scale bar, 10 µm. The area positive for αSma was quantified, normalized to nuclei, and expressed relative to APOE3/3. Pericyte coverage was measured by quantifying the overlapping Ng2 and lectin area, normalized to APOE3/3. Data points represent mean values from individual mice (n = 6 images per mouse, n = 3 mice per genotype). Bars are mean group values ± SEM. P-values were calculated using unpaired two-sample Student’s t-tests. **M.** Schematic of the miBrain model. **N.** Representative images of isogenic APOE3/3 and APOE4/4 miBrains stained for markers of blood vessels (CD144), myofibroblasts (αSMA), and pericytes (NG2). Scale bar, 75 µm. Three-dimensional pericyte coverage was measured by the overlapping NG2 and CD144 volume, normalized to the total CD144 volume, and expressed relative to APOE3/3. The area positive for αSMA was quantified, normalized to nuclei, and expressed relative to APOE3/3. Data points represent mean values from individual miBrains (n = 6 images per miBrain, n = 6 miBrains per genotype). Bars are mean group values ± SEM. P-values were calculated using unpaired two-sample Student’s t-tests.

To generate a single-nuclei transcriptomics atlas of human cerebrovasculature from APOE3/3 and APOE4 carriers, we integrated three previously published snRNA-seq datasets of the postmortem human brain^11,55,56^ (Figure 1A). These studies were selected for their inclusion of *APOE* genotype information and complementary strategies to enrich vascular cell populations that are typically underrepresented in single-cell transcriptomic datasets. To minimize confounding variables, we performed unbiased propensity score matching independently within each dataset to select a final cohort of APOE4 carriers and non-carriers matched for age, sex, AD status, and the number of vascular cell nuclei contributed per individual (Extended Data 1A, 1B). Vascular cells from these matched individuals were harmonized to generate an integrated cerebrovascular atlas (Figure 1B; Extended Data 1C), capturing diverse vascular subtypes across individuals and providing a comprehensive foundation for downstream analyses. After processing and quality control, the atlas consisted of ~64,000 nuclei from 220 individuals (32,396 APOE3/3 (n = 110; F=57, M=53) and 31,673 APOE4 (n = 110; F=53, M=57)) (Extended Data 1A, 1B). Clustering based on canonical cell-type-specific marker gene expression identified distinct populations of the major cerebrovascular cell types: astrocytes (*AQP4*), endothelial cells (*PECAM1*), pericytes (*PDGFRB*), and smooth muscle cells (SMCs) (*MYH11*) (Figure 1B, Extended Data 1D, Supplementary Table S1).

### APOE4 alters vascular cell composition in the human brain

Previous transcriptomic studies of the APOE4 vasculature primarily focused on differential gene expression, with limited statistical power to detect changes in cell type abundance^57^. By harmonizing multiple datasets into a single, integrated atlas, we were able to overcome these limitations and robustly assess whether APOE4 alters the cerebrovascular cell-type composition. We performed differential abundance analysis across all vascular cell types using MiloR, a k-nearest neighbor graph-based approach designed to detect changes in non-discrete cell populations that occur along differentiation or developmental trajectories^58^. Given the plasticity of vascular cell states and the absence of consistent markers to clearly delineate vascular subtypes^59^, this approach is well-suited for capturing abundance differences across transitional populations. Using MiloR, we identified 148 cell neighborhoods with significant differential abundance between APOE4 carriers and APOE3/3 individuals (spatial FDR < 0.05) (Supplementary Table S2).

### Pericyte loss in APOE4 carriers coincides with the emergence of atypical non-vascular SMCs

To examine the directionality of APOE4-associated changes in vascular cell composition, we plotted the fold changes of significantly altered neighborhoods for each cell type (Figure 1C). APOE4 carriers showed a significant reduction of pericytes (0.36-log-fold decrease, p = 0.006) and a significant increase of SMCs (0.75-log-fold increase, p = 0.0001) compared to APOE3/3 individuals. In contrast, astrocytes and endothelial cells exhibited no significant net changes in abundance (1.1-log-fold decrease in astrocytes, p = 0.50; 0.11-log-fold increase in endothelial cells, p = 0.42), leading us to focus our analysis on mural cells.

Age-related cognitive decline is associated with hippocampal cerebrovascular degeneration and elevated cerebrospinal fluid (CSF) levels of the pericyte injury marker soluble PDGFRβ (sPDGFRβ)^31,60,61^. APOE4 further accelerates cognitive decline and increases CSF sPDGFRβ in humans^25^, suggesting that the reduced pericyte abundance we observed in APOE4 carriers may reflect pericyte injury or a cell-state transition. To directly assess APOE4-associated pericyte loss, we performed NG2 immunostaining on postmortem human hippocampal sections. APOE4 carriers exhibited a significant reduction in NG2-positive pericyte coverage along hippocampal vasculature compared to age-matched APOE3/3 individuals (0.21 ± 0.042-fold change, corresponding to a 79% decrease relative to APOE3/3, p = 0.0008, Figure 1D). We next asked whether pericyte loss corresponded with the increase in SMCs revealed by our differential abundance analysis. Human postmortem staining revealed significantly increased αSMA immunoreactivity in the APOE4 hippocampus compared to APOE3/3 controls (1.97 ± 0.35-fold increase, p = 0.0141, Figure 1E). However, while αSMA positivity was primarily confined to large vessels in APOE3/3 hippocampi, APOE4 carriers displayed abundant αSMA immunoreactivity throughout the parenchyma (Figure 1E). Together, these findings indicated that APOE4 carriers exhibit both pericyte loss and the emergence of atypical non-vascular αSMC cells, consistent with a mural cell phenotypic shift. This prompted us to further investigate whether APOE4 alters mural cell identity at the transcriptional level.

### A myofibroblast-like mural cell population is enriched in the APOE4 brain

To investigate APOE4-associated transcriptional changes in mural cells, we subclustered the original pericyte and SMC populations. Consistent with previous studies^55,56^, we identified two pericyte subtypes (Pericyte_1 and Pericyte_1) and two SMC subtypes (SMC_1 and SMC_2) (Figure 1F, Extended Data 1E, Supplementary Table S3). We then performed differential gene expression analysis between APOE4 carriers and APOE3/3 homozygotes within each mural cell subtype (Extended Data 1F, Supplementary Table S4) and employed gene set enrichment analysis to identify dysregulated pathways. Among all mural cell subtypes, SMC_2 showed the greatest impact of APOE4, with 40 dysregulated pathways, followed by Pericyte_1 (11 pathways), SMC_1 (7 pathways), and Pericyte_2 (5 pathways) (FDR < 0.05; Figure 1G, Supplementary Table S5).

Because SMC_2 exhibited the most extensive transcriptional changes, we hypothesized that transcriptional changes in this cluster may underlie the non-vascular αSMA staining pattern observed in the APOE4 postmortem human brain. Pathway enrichment analysis revealed that the most upregulated pathways in APOE4 SMC_2 cells were related to cellular contraction (e.g., muscle contraction, RHO GTPase signaling) and extracellular matrix (ECM) production (e.g., elastic fiber formation, ECM proteoglycans) (Figure 1H). To account for intra-individual cellular correlations, we confirmed upregulation of contraction- and ECM-related pathways in APOE4 SMC_2 cells using a pseudobulk approach (FDR < 0.05) (Extended Data 1G, Supplementary Table S6, S7). SMCs are classically described as adopting either a *contractile state* (high expression of *ACTA2* and *TAGLN* and low ECM gene expression), or a *synthetic state* (low contractile gene expression and high levels of ECM components, including *FN1* and various *collagens*)^62–64^. To determine whether the APOE4 SMC_2 population reflects an expansion of these canonical states or a distinct phenotype with simultaneous activation of both programs, we calculated contraction and ECM gene signature scores for mural cells in our cerebrovascular atlas using established marker genes (Extended Data 1H). Consistent with the pathway analysis, both contraction and ECM signatures were significantly upregulated in SMC_2 cells from APOE4 carriers (contraction score: 1.37 ± 0.10-fold increase, p = 0.0343; ECM score: 1.51 ± 0.10-fold increase, p = 0.0113; Extended Data 1I). Importantly, the proportion of SMC_2 cells co-expressing both ECM and contractile signatures was significantly higher in APOE4 carriers (1.33 ± 0.07-fold increase, p = 0.0321; Extended Data 1I).

The concurrent expression of both signatures is a hallmark of *myofibroblasts*, the principal effector cells of tissue fibrosis^65–70^. Although myofibroblasts have been extensively studied in peripheral organs^71–74^, they have also been reported in the brain following traumatic injury or ischemic events^75,76^, where some are thought to arise from pericytes that detach from the vasculature^75^. To determine whether the APOE4-enriched SMC_2 population resembles myofibroblasts, we performed unbiased cell-type annotation using EnrichR with the CellMarker database^77–79^. Myofibroblasts were identified as the top-matching cell type for the APOE4 SMC_2 gene signature (Figure 1I, Supplementary Table S8). Consistent with this annotation, myofibroblast gene activity scores were significantly higher in SMC_2 cells from APOE4 carriers compared with APOE3/3 individuals (1.39 ± 0.41-fold increase, p = 0.0436; Extended Data 1J).

To determine whether myofibroblasts are selectively enriched in APOE4 carriers, we performed differential abundance analysis of mural cell clusters using DAseq. This analysis revealed a distinct subregion within the SMC_2 cluster that was significantly enriched in APOE4 individuals and spatially colocalized with the peak of ECM and contractile gene co-expression (Figure 1J), a finding independently validated with miloR analysis (Extended Figure 2A). Differential gene expression and pathway enrichment analyses further showed that this APOE4-enriched subregion upregulated genes involved in cell–substrate junctions, ECM organization, and contractile fibers (e.g., *FN1*, *COL3A1*, *ACTA2*, *COL8A1*, *TGFβ1I1*), hallmark features of a myofibroblast state^80^. In contrast, the remaining SMC_2 cells expressed genes linked to vascular stability (e.g., *FLT1*, *EPAS1*, *TIMP3*, *ADAMTS9*) and downregulated cell migration pathways, consistent with a more quiescent, vessel-associated phenotype (Extended Data 2B, C, Supplementary Table S9, S10). Unbiased annotation of this enriched subregion again identified myofibroblast as the top-matching cell type (Extended Data 2D, Supplementary Table S11). Together, the transcriptional profile of APOE4 SMC_2 cells, the results of unbiased cell-type annotation and differential abundance analyses, and the presence of non-vascular parenchymal αSMA staining in postmortem brain tissue strongly support that the APOE4-enriched SMC_2 subpopulation represents myofibroblasts.

Previous studies have shown that cerebrovascular degeneration in APOE4 carriers occurs independently of amyloid and tau pathology^25^, but the mechanisms by which APOE4 drives cerebrovascular degeneration remain unclear. We therefore asked whether the myofibroblast population that we identified arises independently of AD pathology and could represent an upstream driver of APOE4-mediated vascular degeneration. We first analyzed a sub-cohort of non-AD individuals with and without APOE4. For each individual, we quantified the proportion of myofibroblasts and modeled these values using a binomial generalized linear model, adjusting for APOE4 genotype, age, sex, and dataset origin (Extended Data 2E). Model diagnostics confirmed a good fit with no significant violations of assumptions. APOE4 emerged as the strongest predictor of myofibroblast abundance (odds ratio = 5.51 ± 0.28, p < 0.0001), and even in the absence of AD, carriers exhibited significantly higher myofibroblast proportions than APOE3/3 individuals (19.80 ± 2.13% increase, p < 0.0001; Figure 1K, Extended Data 2E), suggesting that APOE4 itself promotes myofibroblast accumulation.

To further test whether APOE4 promotes myofibroblast formation in the absence of AD or other cerebrovascular pathology, we analyzed aged APOE targeted replacement (TR) mice in which the murine *Apoe* gene is replaced with human *APOE3/3* or *APOE4/4* alleles. Bulk RNA-seq data^81^ from cerebral cortices of APOE3/3, APOE3/4, and APOE4/4 TR mice were scored for myofibroblast gene activity using GSVA with marker genes from our human cerebrovascular atlas. APOE4 gene dosage was positively correlated with myofibroblast gene activity (pcc = 0.25, p = 0.038; Extended Data 2F). Immunofluorescence of hippocampal and cortical vessels further validated these findings. While both genotypes exhibited strong αSMA expression in large cerebral vessels (Extended Data 2G), APOE4/4 mice displayed a significant reduction in pericyte coverage of small vessels (< 6 µm) compared with APOE3/3 controls (0.80 ± 0.044-fold change; 20% decrease; p = 0.0189; Figure 1L). This pericyte loss coincided with increased αSMA immunoreactivity in cells proximal to small vessels and within the parenchyma, consistent with a myofibroblast identity (1.74 ± 0.21-fold increase; p = 0.0264; Figure 1L). These results indicate that APOE4 promotes the induction of non-vascular myofibroblasts in aged mice in the absence of other AD genetic factors.

Finally, we assessed whether APOE4 is sufficient to induce myofibroblasts in a human context using miBrains, a 3D human brain tissue derived from isogenic APOE3/3 or APOE4/4 iPSCs differentiated into the six major brain cell types^82,83^ (Figure 1M). Consistent with our human and mouse findings, compared to isogenic APOE3/3 miBrains, APOE4/4 miBrains exhibited a marked reduction in pericyte coverage (0.43 ± 0.056-fold change; 57% decrease; p < 0.0001), accompanied by increased non-vascular αSMA immunoreactivity (3.80 ± 0.43-fold increase; p < 0.0001; Figure 1N). Together, these results demonstrate that APOE4 is sufficient to promote pericyte loss and the emergence of non-vascular myofibroblasts, recapitulating the transcriptional and morphological features observed in human and mouse APOE4 brains.

### APOE4 promotes a pericyte-to-myofibroblast transition

**Figure 2.**
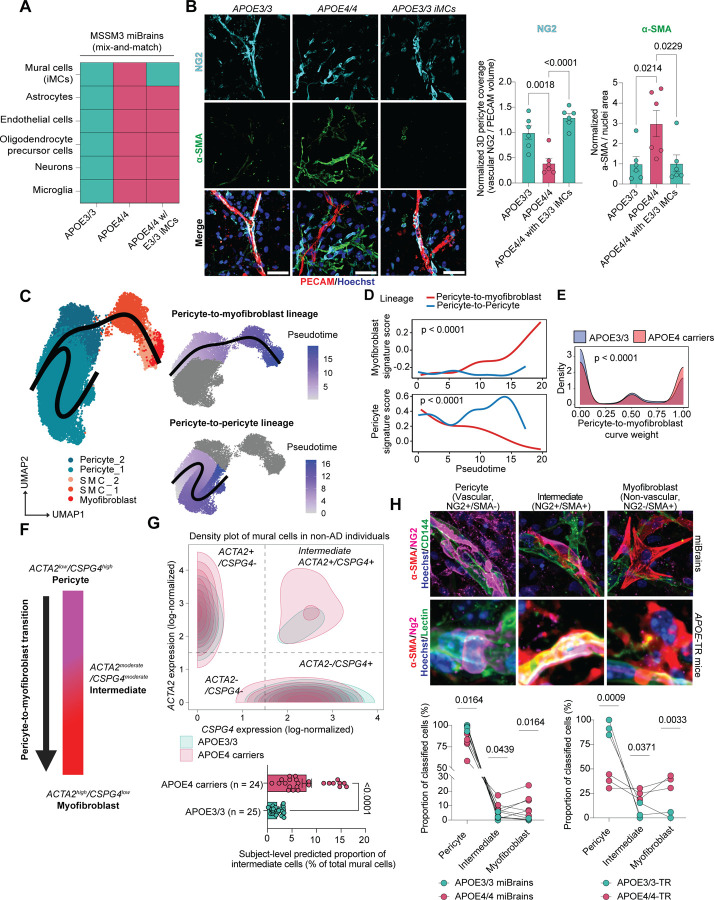
APOE4 promotes a pericyte-to-myofibroblast transition. **A.** Schematic of the hybrid genetic experiment in which APOE3/3 iPSC-derived mural cells (iMCs) were integrated into an APOE4/4 miBrain. **B.** Representative images of isogenic APOE3/3, APOE4/4, and APOE4/4 miBrains integrated with APOE3/3 iMCs stained for PECAM, αSMA, and NG2. Scale bar, 50 µm. Three-dimensional pericyte coverage was measured by the overlapping NG2 and CD144 volume, normalized to the total CD144 volume, and expressed relative to APOE3/3. The area positive for αSMA was quantified, normalized to nuclei, and expressed relative to APOE3/3. Data points represent mean values from individual miBrains (n = 6 images per miBrain, n = 6 miBrains per genotype). Bars are mean group values ± SEM. P-values were calculated using a one-way ANOVA with Dunnett’s multiple comparisons test. **C.** Pseudotime analysis of the mural cell subclusters identified in Figure 1 using the slingshot R package. **D.** Generalized additive models (GAMs) were fit along each lineage using curated myofibroblast- and pericyte-associated gene signatures (see Methods) to validate the Slingshot-inferred trajectories. Signature score trajectories were then compared between lineages by computing the area under the curve (AUC) across pseudotime, and significance was assessed using a permutation-based p-value. **E.** Lineage bias between APOE4 carriers and APOE3/3 individuals. Distributions of Slingshot-derived curve weights were plotted for each lineage, stratified by APOE genotype. A value of 0 indicates a cell is exclusive to the pericyte-to-pericyte lineage. A value of 1 indicates a cell is exclusive to the pericyte-to-myofibroblast lineage. The p-value was calculated using the differentiationTest function in the condiments R package (see Methods). **F.** Schematic of *ACTA2* and *CSPG4* gene expression gradient during the pericyte-to-myofibroblast transition. **G.** Density plot of mural cells in non-AD individuals stratified by *ACTA2* and *CSPG4* expression. Cutoffs for gene expression were set at a log-normalized count value of 1.5. Subject-level predicted proportion of intermediate cells (*ACTA2+/CSPG4+*) in non-AD individuals was plotted as estimated by the binomial generalized linear model described in Extended Data 4B–C. Data points represent individuals (n = 25 APOE3/3, n = 24 APOE4 carriers). Bars are group means ± SEM. P-values are derived from the binomial GLM. **H.** Representative images of the pericyte-to-myofibroblast transition in APOE4/4 miBrains and TR-mice. APOE3/3 and APOE4/4 miBrains and TR-mice were stained for a vascular marker (CD144/lectin), αSMA, and NG2. Cells were classified into pericyte (vascular NG2+/ αSMA-), intermediate (NG2+/ αSMA+), or myofibroblast (nonvascular NG2-/ αSMA+) categories using intensity-based thresholds in CellProfiler. The number of cells in each category was normalized to the total number of classified cells. Data points represent mean values from individual miBrains or mice (n = 6 images per miBrain/mouse, n = 6 miBrains and n = 3 mice per genotype). P-values represent comparisons between genotypes for each cell state. P-values were calculated using a two-way repeated measures ANOVA with Geisser-Greenhouse correction and Tukey’s multiple comparisons test.

Having identified a robust APOE4-associated myofibroblast phenotype across three model systems, we next sought to determine whether this phenotype arises within mural cells or requires interaction with other APOE4-expressing cell types. To test this, we performed a hybrid genetic mixing experiment in which isogenic APOE3/3 mural cells (iMCs) were selectively integrated into APOE4/4 miBrains (Figure 2A). As expected, APOE4/4 miBrains exhibited significantly reduced pericyte coverage and increased αSMA immunoreactivity compared to isogenic APOE3/3 controls (pericyte coverage: 0.39 ± 0.10-fold change, corresponding to 61% decrease relative to APOE3/3, p = 0.0018; αSMA: 2.99 ± 0.65-fold increase, p = 0.0214; Figure 2B). Strikingly, replacing APOE4/4 iMCs with isogenic APOE3/3 iMCs reversed these phenotypes, leading to a significant reduction in αSMA-positive cells and restoration of NG2-positive cell coverage along the vasculature (pericyte coverage: 1.60 ± 0.016-fold increase relative to APOE4/4, p < 0.0001; a-SMA: 0.34 ± 0.14-fold change, corresponding to a 66% decrease relative to APOE4/4, p = 0.0229; Figure 2B). These findings indicated that APOE4 expression in mural cells is necessary for pericyte loss and myofibroblast accumulation. We repeated this experiment with iMCs derived from two additional isogenic iPSC lines from an AD and non-AD individual with reciprocal gene editing strategies (Non-AD E3→E4 [ADRC line] and AD E4→E3 [sAD line]) (Extended Data 3A, B) and found similar results (Extended Data 3C, D). To assess whether APOE4 mural cells are sufficient to induce myofibroblasts, we examined iMC monocultures. Immunostaining showed significantly higher αSMA and fibronectin levels in APOE4/4 iMCs compared to isogenic APOE3/3 controls across all donor lines (Extended Data 3E–G). Taken together, these findings from both APOE-TR mice and our iPSC models point to a causal role for APOE4 mural cells in the expansion of a myofibroblast population.

We next investigated how APOE4 mural cells generate the myofibroblast population. In multiple organ systems, including the brain, pericytes differentiate into myofibroblasts following injury^71,75^. A pericyte-to-myofibroblast transition (PMT) would explain the observed correlation between myofibroblast expansion and pericyte loss (Figure 1D, E, L, N). To explore this possibility, we performed pseudotime analysis of our human cerebrovascular atlas. This revealed two distinct pericyte-derived trajectories: one first terminating in the APOE4-enriched myofibroblast population (pericyte-to-myofibroblast lineage) and another terminating in pericytes (pericyte-to-pericyte lineage) (Figure 2C). Gene signature scoring confirmed that the first trajectory was characterized by strong myofibroblast gene activity (AUC = 2.45, p < 0.0001) and the loss of pericyte identity (AUC = −4.18, p < 0.0001) (Figure 2D). To determine whether *APOE* genotype influences lineage conversion, we calculated curve weights for both trajectories. APOE4 mural cells were significantly enriched along the pericyte-to-myofibroblast trajectory, whereas APOE3/3 cells preferentially aligned with the pericyte-to-pericyte trajectory (Figure 2E).

In vivo, the pericyte-to-myofibroblast transition is reported to occur as a gradual continuum, rather than a binary switch, with cells progressively shifting from a pericyte to a myofibroblast identity. This process includes an intermediate state in which cells co-express *CSPG4* (pericyte marker) and *ACTA2* (SMC/myofibroblast marker) (Figure 2F)^71,84^. We reasoned that if APOE4 promotes a PMT, APOE4 carriers should show not only pericyte loss and a gain of myofibroblasts, but also an enrichment of these intermediate *CSPG4+/ACTA2+* mural cells. To test this, we identified double-positive cells in our cerebrovascular atlas using defined gene expression cutoffs. We restricted the analysis to non-AD individuals to isolate the effect of *APOE* genotype. APOE4 carriers exhibited a higher abundance of double-positive *CSPG4+/ACTA2+* cells compared to APOE3/3 individuals (4.60-fold increase, p < 0.0001; Figure 2G, Extended Data 4A). To confirm this enrichment, we modeled this proportion of intermediate cells per individual using a binomial generalized linear model, adjusted for *APOE* genotype, age, sex, and dataset origin (Extended Data 4B). Model diagnostics indicated a good fit, with no significant violations of model assumptions (Extended Data 4B). APOE4 genotype emerged as the strongest predictor of *CSPG4+/ACTA2+* mural cell abundance (odds ratio = 5.51 ± 0.10, *p* < 0.0001; Extended Data 4C). Even in the absence of AD, APOE4 carriers had a significantly higher proportion of intermediate cells than age- and sex-matched APOE3/3 individuals (5.98 ± 0.76% increase, p < 0.0001; Figure 2G), supporting an APOE4-driven pericyte-to-myofibroblast transition.

To determine whether this intermediate state is also present in our experimental models, we quantified mural cells in miBrains and APOE-TR mice as pericytes (vascular NG2+/αSMA–), intermediate cells (NG2+/αSMA+), or myofibroblasts (non-vascular NG2–/αSMA+). To avoid misclassification, analyses were restricted to small vessels (< 6 µm) lacking SMCs, focusing on non-vascular αSMA+ cells. Consistent with our atlas findings, APOE4/4 miBrains and APOE4/4-TR mice exhibited a significant reduction in pericyte abundance (miBrains: 80.55 ± 4.90% APOE4/4 vs. 97.68 ± 1.08% APOE3/3, p = 0.0116; APOE-TR mice: 37.56 ± 4.09% APOE4/4-TR vs. 91.93 ± 4.46% APOE3/3-TR, p = 0.0009; Figure 2H). This decrease coincided with a significant increase in APOE4/4 myofibroblast abundance that was absent in isogenic APOE3/3 control (miBrains: 11.44 ± 3.14% APOE4/4 vs. 0.33 ± 0.22% APOE3/3, p = 0.0164; APOE-TR mice: 37.68 ± 3.72% APOE4/4 vs. 1.96 ± 1.96% APOE3/3, p = 0.0033; Figure 2H). Notably, in APOE4 mice and miBrains we also observed cells along small cerebral vessels strongly co-expressing αSMA and NG2, reflective of an intermediate transition state associated with the PMT (miBrains: 8.02 ± 2.25% APOE4/4 vs. 1.99 ± 0.88% APOE3/3, p = 0.0439; APOE-TR mice: 24.76 ± 3.28% APOE4/4 vs. 6.11 ± 4.72% APOE3/3, p = 0.0371; Figure 2H). To further validate the occurrence of a pericyte-to-myofibroblast transition in our experimental models, we quantified the average distance of each cell type from the vasculature in both APOE4/4 miBrains and APOE4/4-TR mice. In both model systems, vascular distance increased in a stepwise manner from pericytes to intermediate cells to myofibroblasts, supporting a model in which pericytes detach from vessels and migrate into the parenchyma while acquiring myofibroblast characteristics (APOE4/4 miBrains: R^2^ = 0.53, p = 0.0009; APOE4/4-TR mice: R^2^ = 0.81, p = 0.0028; Extended Data 4D). Taken together, these results support that APOE4 promotes a stepwise pericyte-to-myofibroblast transition in both human and *in vivo* models.

Finally, we examined the APOE4-induced PMT in iMC monocultures. Using CIBERSORTx^85^ deconvolution of a bulk RNA-seq dataset^47^, we mapped iMCs onto mural cell subclusters from our cerebrovascular atlas (Extended Data 4E). Without stratifying by *APOE* genotype, we found that iMCs transcriptionally align most with the Pericyte_1 cluster compared to other mural cell subtypes (Extended Data 4F). However, investigating cell type identity scores between genotypes revealed two significant shifts: a loss of Pericyte_1 identity and a gain of myofibroblast identity in APOE4/4 iMCs relative to APOE3/3 (Pericyte_1: −0.36 ± 0.078 logFC, adjusted p = 0.0473; myofibroblast: 0.53 ± 0.063 logFC, adjusted p = 0.0116; Extended Data 4G). Differential expression analysis further confirmed upregulation of canonical myofibroblast genes in APOE4/4 iMCs (Extended Data 4H, I, Supplementary Table S12, S13). Collectively, these results across human tissue, iPSC-derived models, and APOE-TR mice demonstrate that APOE4 in mural cells is sufficient to drive a pericyte-to-myofibroblast transition.

### Myofibroblast-derived fibronectin increases amyloid accumulation in APOE4 models

**Figure 3.**
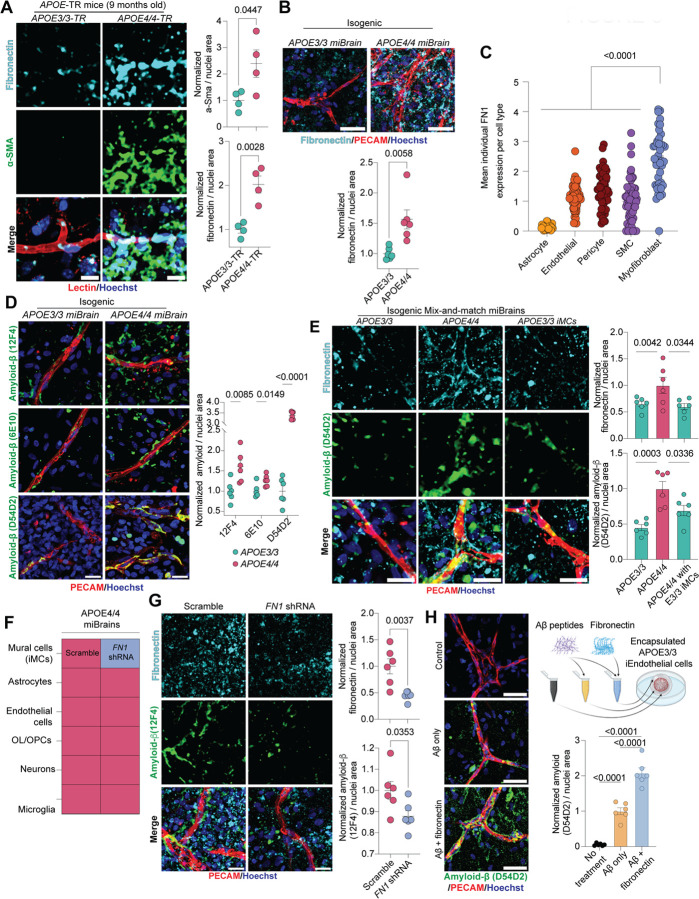
Myofibroblast-derived fibronectin drives amyloid accumulation in APOE4 models. **A.** Representative images of small vessels in the corpus callosum of APOE3/3-TR and APOE4/4-TR mice stained for lectin, αSma, and fibronectin. Scale bar, 10 µm. **B.** Representative images of APOE3/3 and APOE4/4 miBrains stained for PECAM and fibronectin. Scale bar, 50 µm. **C.** Quantification of *FN1* expression across cell types in the cerebrovascular atlas. *FN1* expression was aggregated per cell type for each individual. Individuals missing more than one cell type were excluded from the analysis. Data points represent individuals (n = 57). Bars are group means ± SEM. P-values were calculated using a repeated measures mixed-effects analysis with the Geisser-Greenhouse correction and Dunnett’s multiple comparisons test. **D.** Representative images of isogenic pairs of APOE3/3 and APOE4/4 miBrains stained for three amyloid antibodies targeting different epitopes of the amyloid-β peptide. Scale bar, 25 µm. **E.** Representative images of isogenic APOE3/3, APOE4/4, and APOE4/4 miBrains integrated with APOE3/3 iMCs stained for PECAM, fibronectin, and amyloid (D54D2). Scale bar, 25 µm. Data represent two independent experiments. In the first experiment, APOE3/3 and APOE4/4 were compared using an unpaired two-sample Student’s t-test. In the second experiment, APOE4/4 was compared to APOE4/4 with APOE3/3 iMCs using an unpaired two-sample Student’s t-test. P-values shown reflect these independent comparisons. For clarity, only the APOE4/4 group from the second experiment is shown. Representative images are from the corresponding experiment in each condition. **F.** Schematic of *FN1* knockdown experiment in APOE4/4 miBrains. **G.** Representative images of APOE4/4 miBrains integrated with either iMCs transduced with scramble shRNA or shRNA targeting *FN1* stained for fibronectin and amyloid (12F4). Scale bar, 25 µm. **H.** Treatment of encapsulated APOE3/3 iPSC-derived endothelial cells (iECs) with fibronectin and amyloid mixtures. Scale bar, 50 µm. For the control group, iECs were left untreated. For the experimental groups, iECs were treated with either 20 nM amyloid-β 1–40 and 1–42 or 20 nM amyloid-β 1–40 and 1–42 mixed with 50 ng/mL fibronectin for 96 hrs. iECs were then fixed and stained for amyloid (D54D2). For all panels, the area positive for αSMA, fibronectin, or amyloid immunoreactivity was quantified, normalized to nuclei, and expressed relative to APOE3/3 (panels B-D, H) or APOE4/4 (panels E, G). Data points represent mean values from individual miBrains (n = 6 images per miBrain, n = 6 miBrains per condition) or mice (n = 6 images per mouse, n = 4 mice per genotype). Bars are mean group values ± SEM. Unless stated otherwise, p-values were calculated using unpaired two-sample Student’s t-tests (panels B-D, G) or a one-way ANOVA with Dunnett’s multiple comparisons test (panel H).

Myofibroblasts are key mediators of fibrosis, functioning as major sources of extracellular matrix (ECM) proteins, particularly collagens and fibronectin (*FN1*)^86^. Excessive ECM deposition by myofibroblasts leads to pathological tissue remodeling and organ dysfunction^87^. Recent studies have identified genetic variants in *FN1* associated with cognitive resilience in APOE4 carriers^50^, suggesting that altered fibronectin biology may influence disease outcomes in APOE4 carriers. We therefore examined whether APOE4-associated myofibroblasts promote fibronectin deposition in the brain vasculature. In APOE4/4-TR mice, fibronectin immunoreactivity was significantly increased along small vessels compared to age- and sex-matched APOE3/3-TR controls (2.02 ± 0.19-fold increase, p = 0.0028), coinciding with elevated αSMA staining (2.39 ± 0.52-fold increase, p = 0.0447; Figure 3A). Notably, most of the fibronectin deposition in APOE4/4-TR mice occurred near αSMA+ regions (72.66 ± 1.90%, p = 0.0013, Extended Data 5A), implicating myofibroblasts as a causal source of vascular fibrosis. Consistent with these findings, APOE4/4 miBrains also exhibited increased fibronectin accumulation along the vasculature relative to isogenic APOE3/3 miBrains (1.56 ± 0.16-fold increase, p = 0.0058; Figure 3B).

Myofibroblasts were the largest contributor of *FN1* expression among individuals from our cerebrovascular atlas (Figure 3C). In the central nervous system, perivascular fibroblasts are also a major source of fibronectin and contribute to fibrotic scar formation in response to brain injury^88,89^. To further confirm that APOE4-myofibroblasts are primarily responsible for *FN1* upregulation, we analyzed perivascular fibroblasts from the original snRNA-seq datasets corresponding to the same subjects included in our cerebrovascular atlas. We found that the myofibroblasts identified in this study exhibited significantly (p < 0.0001) higher *FN1* expression compared to fibroblasts (Extended Data 5B), and that *APOE* genotype had no significant (FDR > 0.05) effect on ECM-related pathways in fibroblasts (Supplementary Tables S14, S15), further highlighting myofibroblasts as the causal cell type in our study.

To assess how mural cell *FN1* expression relates to clinical characteristics of AD, we analyzed our cerebrovascular atlas using the Haney (2024) dataset^11^, which includes extensive clinical annotations. We focused on individuals with an AD diagnosis and pseudobulked mural cell *FN1* expression for each individual to specifically examine *FN1* within the context of the pericyte-to-myofibroblast transition. Consistent with our earlier findings, APOE4 carriers had significantly higher mural cell *FN1* expression compared to APOE3/3 individuals (1.30 ± 0.11-fold increase, p = 0.0294; Extended Data 5C). Supporting a role for fibronectin in AD progression, mural cell *FN1* expression negatively correlated with age of AD diagnosis (r = −0.53, p = 0.036, Extended Data 5B). We also derived a global pathology score for each individual by averaging their total amyloid plaque, neurofibrillary tangle, Lewy body, and cerebral infarct scores reported in the original dataset. Individuals in the highest quartile of global pathology had significantly elevated mural cell *FN1* expression compared to the remaining individuals (1.33 ± 0.11-fold increase relative to the low pathology group, p = 0.0114; Extended Data 5C), suggesting a link between APOE4-driven fibronectin deposition and AD pathology.

To investigate the causal role of fibronectin in AD pathology, we leveraged the miBrain system. Because APOE4 carriers are highly susceptible to vascular amyloid deposition and fibronectin can directly bind amyloid^90–92^, we focused on perivascular amyloid-β accumulation. To first validate the miBrain as a model for this pathology, we immunostained isogenic APOE3/3 and APOE4/4 miBrains using three separate antibodies each targeting distinct amyloid-β epitopes. Consistent with observations in APOE4 carriers, APOE4/4 miBrains showed significantly increased amyloid-β immunoreactivity along the vasculature with all three antibodies compared to isogenic APOE3/3 controls (12F4: 1.61 ± 0.16-fold increase, p = 0.0085; 6E10: 1.26 ± 0.056-fold increase, p = 0.0149; D54D2: 3.38 ± 0.074-fold increase, p < 0.0001; Figure 3D).

Our previous results indicate that APOE4 mural cell-derived myofibroblasts are the primary source of increased fibronectin deposition in APOE4/4-TR mice (Extended Data 5B). To directly test this, we swapped APOE4/4 mural cells to APOE3/3 in an APOE4/4 miBrain, an approach that previously reversed APOE4-associated myofibroblast phenotypes (Figure 2B).

Remarkably, this single swap led to a significant reduction in fibronectin protein accumulation to similar levels as the APOE3/3 baseline (0.60 ± 0.058-fold change, corresponding to a 40% decrease relative to APOE4/4, p = 0.0344; Figure 3E), demonstrating that APOE4 vascular fibrosis is driven by myofibroblasts. These results were reproduced with isogenic mural cells derived from two different donors (with and without AD) (Extended Data 5D). If fibronectin causally contributes to amyloid-β pathology, then reducing myofibroblast-derived fibronectin should also lower amyloid accumulation. Consistent with this, APOE4/4 miBrains containing APOE3/3 iMCs displayed significantly reduced vascular amyloid deposition compared to all APOE4/4 controls (0.69 ± 0.073-fold change, corresponding to a 31% decrease relative to APOE4/4, p = 0.0336; Figure 3E).

To determine whether the reduction in amyloid pathology observed when APOE3/3 iMCs replaced APOE4/4 iMCs in the miBrain was mediated by decreased fibronectin, we transduced APOE4/4 iMCs with a shRNA targeting *FN1* or a scramble control. qRT-PCR of iMC monocultures confirmed robust *FN1* knockdown (0.037 ± 0.0018-fold change, corresponding to a 96.3% decrease relative to scramble, p = 0.0009; Extended Data 5E). FN1 knockdown and control APOE4/4 iMCs were then incorporated into APOE4/4 miBrains to directly assess the impact of fibronectin depletion on amyloid accumulation (Figure 3F). Consistent with our hypothesis, *FN1* knockdown led to markedly reduced fibronectin staining and a concomitant decrease in vascular amyloid deposition compared to scramble controls (fibronectin: 0.44 ± 0.036-fold change, corresponding to a 56% decrease relative to scramble, p = 0.0037; amyloid: 0.88 ± 0.027-fold change, corresponding to a 12% decrease relative to scramble, p = 0.0353; Figure 3G). To further validate the causal link between fibronectin and amyloid accumulation, we treated APOE3/3 iPSC-derived endothelial cells with amyloid alone or an amyloid-fibronectin mixture. Cells exposed to the amyloid-fibronectin mixture exhibited significantly greater amyloid accumulation than those treated with amyloid alone (2.08 ± 0.15-fold increase, p < 0.0001, Figure 3H). Together, these findings demonstrate that fibronectin secreted by APOE4-derived myofibroblasts promotes vascular amyloid accumulation, establishing a direct mechanistic link between myofibroblasts and AD progression in APOE4 carriers.

### Increased TGF-β signaling in the APOE4 brain promotes the pericyte-to-myofibroblast transition

**Figure 4.**
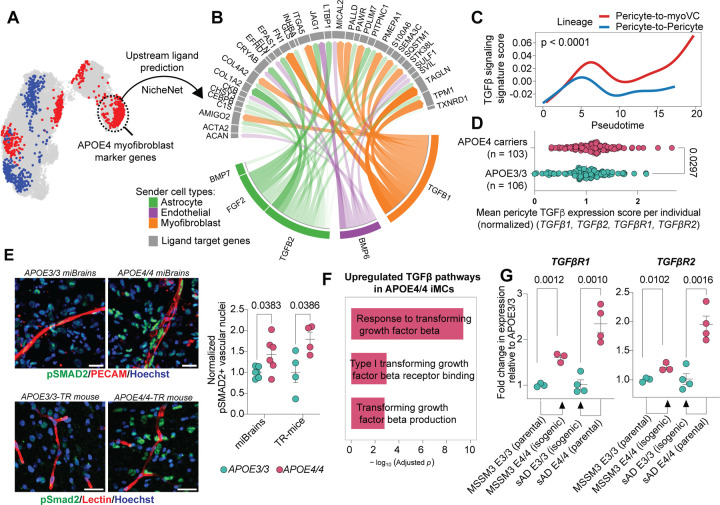
Increased TGF-β signaling in the APOE4 brain drives the pericyte-to-myofibroblast transition. **A.** APOE4 myofibroblast marker genes identified in Extended Data 2B were input into the R package NicheNet. **B.** Cirkos plot of top ligands and their predicted target genes from NicheNet. **C.** A generalized additive model (GAM) was fitted along each lineage using the hallmark TGF-β signaling gene set (see Methods). Signature score trajectories were then compared between lineages by computing the area under the curve (AUC) across pseudotime, and significance was assessed using a permutation-based p-value. **D.** TGF-β expression scores of pericytes from APOE4 carriers and APOE3/3 individuals. A TGF-β module scores were calculated using Seurat’s *AddModuleScore()* function based on the specified genes. The scores from Pericyte 1 and Pericyte 2 cells were then averaged per individual. Data points represent individuals (n = 106 APOE3/3, n = 103 APOE4 carriers). Bars are group medians. P-values were calculated using the Wilcoxon rank-sum test. **E.** Representative images of APOE3/3 and APOE4/4 miBrains and TR-mice stained for a vascular marker (PECAM/lectin) and pSMAD2. Scale bar, 25 µm. Nuclei along the vasculature were classified as pSMAD2+ using an intensity-based threshold in CellProfiler, normalized to the total number of vascular nuclei, and expressed relative to APOE3/3. Data points represent mean values from individual miBrains or mice (n = 6 images per miBrain/mouse, n = 6 miBrains and n = 4 mice per genotype). Bars are mean group values ± SEM. P-values were calculated using unpaired two-sample Student’s t-tests. **F.** Upregulated TGF-β signaling GO pathways in APOE4/4 iPSC-derived mural cells (iMCs). Differentially expressed genes in the iMC bulk RNA-seq dataset (|logFC| > 0.25, adjusted p < 0.05 calculated from limma) were input into ClusterProfiler. The significantly upregulated TGF-β signaling GO pathways (FDR < 0.05) were then extracted for graphical representation. **G.** *TGFβR* expression in isogenic pairs of iMCs from two donor lines. MSSM3 line: FPKM values from iMC bulk RNA-seq dataset normalized to APOE3/3. Data points represent individual bulk RNA-sequencing replicates (n = 3 per genotype). Bars are mean group values ± SEM. Adjusted p-values were calculated using the R package limma to account for multiple comparisons. sAD line: *TGFβR* expression in iMCs via qRT-PCR relative to APOE3/3. Data points represent mean values from individual wells of iMCs (3 qRT-PCR replicates per well, 4 wells per genotype). Bars are mean group values ± SEM. P-values were calculated using unpaired two-sample Student’s t-tests.

Having identified a key role for APOE4-derived myofibroblasts in AD pathology, we next investigated the molecular mechanisms through which APOE4 promotes the PMT. We applied NicheNet^93^, a computational tool that predicts upstream ligands regulating target gene expression, using APOE4 myofibroblast marker genes identified in our human cerebrovascular atlas as input (Figure 4A, Figure 1J). NicheNet identified TGF-β as the top predicted driver of the PMT (Figure 4B, Extended Data 5F, Supplementary Table S16). In agreement with its established role in fibrosis, TGF-β ligands were predicted to regulate multiple myofibroblast-associated genes, including *FN1*, *ACTA2*, and *COL1A2* (Figure 4B, Extended Data 5G). Fibrosis in the CNS can also arise downstream of an interferon-gamma-mediated cascade, particularly in neuroinflammatory diseases such as multiple sclerosis^88^. However, APOE4 myofibroblasts show no change in interferon-gamma signaling at either the gene or pathway level (Supplementary Tables S4–7, S9, S10). These findings suggest that APOE4 cerebrovascular fibronectin and amyloid deposition occur through a TGF-β-dependent, IFN-γ-independent mechanism.

Within our postmortem human cerebrovascular atlas, TGFβ1 was predominantly expressed by myofibroblasts and TGFβ2 by astrocytes, with only modest differences in ligand expression between APOE4 and APOE3/3 individuals (Figure 4B, Extended Data 5H, I). However, TGF-β signature scoring along pseudotime lineages revealed significantly increased TGF-β hallmark gene activity along the pericyte-to-myofibroblast trajectory compared to the pericyte-to-pericyte trajectory (0.479 AUC, p < 0.0001; Figure 4C). Furthermore, pericytes from APOE4 carriers displayed significant upregulation of key TGF-β genes (*TGFβ1, TGFβ2, TGFβR1, TGFβR2*) relative to APOE3/3 controls (1.13-fold increase, IQR: 0.90–1.34, p = 0.0297; Figure 4D). To functionally validate the increase in TGF-β signaling along the APOE4 vasculature, we immunostained miBrains and APOE-TR mice for pSmad2, a canonical marker of TGF-β pathway activation. Consistent with our computational predictions, APOE4/4 miBrains exhibited significantly more pSMAD2+ vascular nuclei compared to APOE3/3 (1.43 ± 0.17-fold increase, p = 0.0383; Figure 4E). Similarly, APOE4/4-TR mice showed significantly more pSmad2 immunoreactive nuclei around cortical microvasculature compared to age-matched APOE3/3-TR mice (1.79 ± 0.17-fold increase, p = 0.0386; Figure 4E).

We next sought to identify the cellular source of increased TGF-β in APOE4 brains. Given our prior finding that APOE4 mural cells are both necessary and sufficient to generate myofibroblasts, we hypothesized that TGF-β pathway activation is intrinsic to APOE4 mural cells. Supporting this, APOE4/4 iMCs in monoculture exhibited significantly higher nuclear SMAD2 intensities compared to isogenic APOE3/3 controls (1.21 ± 0.060-fold increase, p = 0.0104; Extended Data 5J). Moreover, treating APOE3/3 iMCs with recombinant TGFβ1 (50 ng/mL) increased nuclear SMAD2 levels comparable to APOE4/4 cells (1.17 ± 0.016-fold increase, p = 0.0393; Extended Data 5J), indicating that elevated baseline TGF-β signaling in APOE4/4 mural cells accounts for their increased SMAD2 activation.

To explore the mechanism underlying this elevated signaling, we analyzed iMC bulk RNA-seq data. Consistent with our immunofluorescence findings, APOE4/4 iMCs exhibited significant upregulation of TGF-β pathway genes (Figure 4F). We reasoned that enhanced signaling could result from either increased TGF-β ligand production or upregulation of TGF-β receptors. Analysis of TGF-β ligand expression revealed no significant differences between genotypes for *TGFβ1* (1.09 ± 0.062-fold increase in APOE4/4, adjusted p = 0.63; Supplementary Table S12) and *TGFβ3* (1.04 ± 0.022-fold increase in APOE4/4, adjusted p = 0.50; Supplementary Table S12, and even a significant decrease in *TGFβ2* expression for APOE4/4 iMCs compared to isogenic control APOE3/3 (0.47 ± 0.013-fold change, corresponding to a 53% decrease in APOE4/4 relative to APOE3/3, adjusted p = 0.001; Supplementary Table S12). By contrast, both *TGFβR1* and *TGFβR2* were significantly upregulated in APOE4/4 mural cells compared to isogenic APOE3/3 controls (*TGFβR1:* 1.61 ± 0.057-fold increase, adjusted p = 0.0012; *TGFβR2:* 1.22 ± 0.042-fold increase, adjusted p = 0.0102 Figure 4G). We validated these findings in another iMC isogenic pair via qRT-PCR (*TGFβR1*: 2.35 ± 0.20-fold increase, adjusted p = 0.0020; *TGFβR2*: 1.95 ± 0.15-fold increase, adjusted p = 0.0020; Figure 4G). Finally, to test whether receptor upregulation is functionally linked to myofibroblast induction, we inhibited TGF-β signaling using the SB431542 compound. This treatment significantly reduced αSMA and fibronectin expression (αSMA: 0.39 ± 0.033-fold change, corresponding to a 61% decrease relative to APOE4/4, p < 0.0001; fibronectin: 0.76 ± 0.085-fold change, corresponding to a 24% decrease relative to APOE4/4, p = 0.0308; Extended Data 5K). Together, these results indicate that increased expression of TGF-β receptors in APOE4 mural cells drives enhanced TGF-β signaling and promotes the pericyte-to-myofibroblast transition.

### TGF-β inhibition reverses APOE4-driven myofibroblast accumulation, cerebrovascular fibrosis, and amyloid deposition

**Figure 5.**
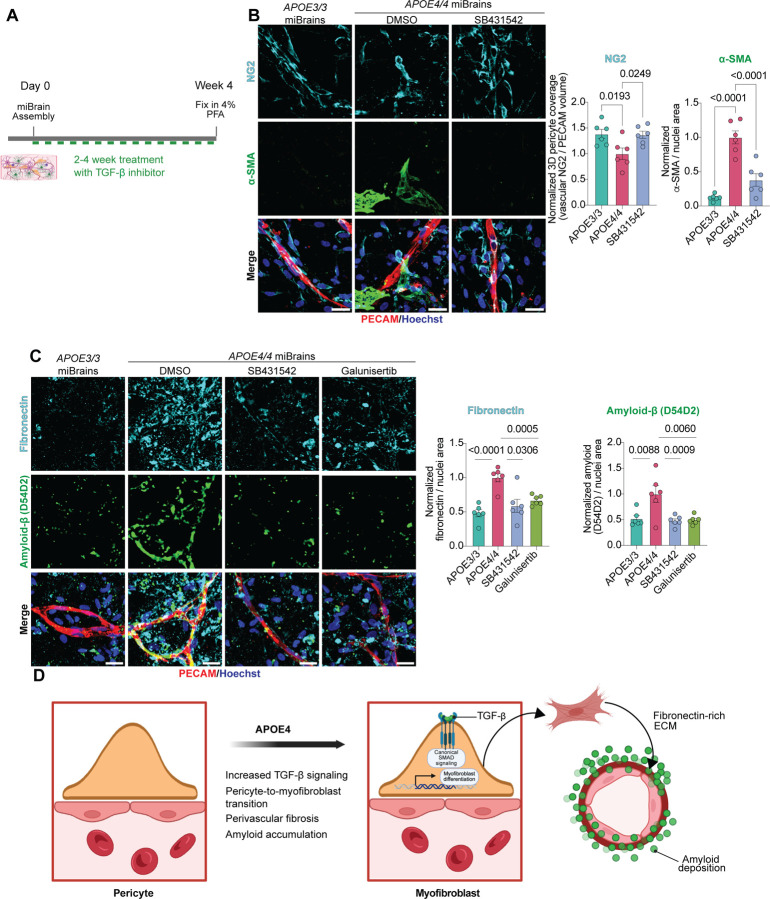
TGF-β inhibition reverses APOE4-driven myofibroblast accumulation, cerebrovascular fibrosis, and amyloid deposition. **A.** Timeline of TGF-β inhibition in miBrains. **B.** Representative images of myofibroblast phenotypes after TGF-β inhibition in miBrains. Scale bar, 25 µm. For the control group, APOE3/3 and APOE4/4 miBrains were treated with a DMSO vehicle for two weeks. For the experimental group, APOE4/4 miBrains were treated with 10 µM SB431542 for two weeks. miBrains were then fixed and stained for PECAM, αSMA, and NG2. Three-dimensional pericyte coverage was measured by the overlapping NG2 and PECAM volume, normalized to the total PECAM volume and expressed relative to APOE4/4. The area positive for αSMA was quantified, normalized to nuclei, and expressed relative to APOE4/4. Data points represent mean values from individual miBrains (n = 6 images per miBrain, n = 6 miBrains per condition). Bars are mean group values ± SEM. P-values were calculated using a one-way ANOVA with Dunnett’s multiple comparisons test. **C.** Representative images of cerebrovascular fibrosis and amyloid deposition in miBrains after TGF-β inhibition. Scale bar, 25 µm. For the control group, APOE3/3 and APOE4/4 miBrains were treated with a DMSO vehicle for 4 weeks. For the experimental group, APOE4/4 miBrains were treated with either 50 µM SB431542 or 50 µM galunisertib for 4 weeks. miBrains were then fixed and stained for PECAM, fibronectin, and amyloid (D54D2). The data represent two independent experiments. In the first experiment, APOE3/3 DMSO, APOE4/4 DMSO, and APOE4/4 treated with galunisertib were compared using a one-way ANOVA with Dunnett’s multiple comparisons test. In the second experiment, APOE4/4 DMSO was compared to APOE4/4 treated with SB431542 using an unpaired two-sample Student’s t-test. P-values shown reflect these independent comparisons. For clarity, only the APOE4/4 DMSO group from the first experiment is shown. Representative images are from the corresponding experiment in each condition. Data points represent mean values from individual miBrains (n = 6 images per miBrain, n = 6 miBrains per condition). Bars are mean group values ± SEM. **D.** Mechanistic model of the TGF-β-mediated pericyte-to-myofibroblast transition in APOE4 carriers.

AD patients exhibit significantly elevated *TGFβ1* mRNA levels that strongly correlate with CAA severity^54^ , and chronic TGF-β overexpression in mice induces cerebrovascular fibrosis and perivascular amyloid accumulation^52,53^. Our findings support a model in which heightened TGF-β signaling in APOE4 brains causes a PMT leading to vascular fibronectin and amyloid deposition. To directly test this mechanism, we inhibited TGF-β signaling in APOE4/4 miBrains using two chemically distinct ALK5 (TGFβR1) inhibitors (SB431452 and Galunisertib) (Figure 5A).

Treatment of APOE4/4 miBrains with the TGF-β inhibitor significantly reduced non-vascular αSMA immunoreactivity and led to a significant increase of pericyte vascular coverage to levels comparable to APOE3/3 controls (αSMA: 0.38 ± 0.091-fold change, corresponding to a 62% decrease relative to APOE4/4, p < 0.0001; pericyte coverage: 1.37 ± 0.072-fold increase relative to APOE4/4, p = 0.0249; Figure 5B), indicating a reversal of the PMT. Given the causal link we established between fibronectin secretion from myofibroblasts and perivascular amyloid accumulation, we next tested whether TGF-β inhibition could reduce both vascular fibrosis and amyloid burden. Consistent with this prediction, treatment of APOE4/4 miBrains with either SB431542 or Galunisertib significantly decreased fibronectin levels (SB431542: 0.59 ± 0.095-fold change, corresponding to a 41% decrease relative to APOE4/4, p = 0.0306; Galunisertib: 0.67 ± 0.030-fold change, corresponding to a 33% decrease relative to APOE4/4, p = 0.0005; Figure 5C). Notably, these reductions in fibronectin were accompanied by significant reductions in amyloid deposition, restoring levels to those seen in APOE3/3 control miBrains (SB431542: 0.48 ± 0.041-fold change, corresponding to a 52% decrease relative to APOE4/4, p = 0.0009; Galunisertib: 0.49 ± 0.034-fold change, corresponding to a 51% decrease relative to APOE4/4, p = 0.0060; Figure 5C). Together, these findings demonstrate that APOE4 promotes a pericyte-to-myofibroblast transition through upregulation of TGF-β receptor signaling, driving vascular fibrosis and fibronectin-dependent amyloid accumulation, and that pharmacological inhibition of this pathway reverses these pathological phenotypes in human brain tissue (Figure 5D).

## Discussion

Overall, the results presented here reveal that elevated TGF-β signaling drives a pericyte-to-myofibroblast transition in the APOE4 brain. This leads to a loss of pericyte coverage and fibrosis of the APOE4 vasculature, with fibronectin accumulation directly promoting perivascular amyloid deposition. Pharmacologically inhibiting TGF-β signaling in APOE4 human brain tissue reverses the pericyte-to-myofibroblast transition and attenuates cerebrovascular pathology. These results establish a causal link between TGF-β and cerebrovascular dysfunction in APOE4 carriers, which may influence AD progression in this large patient population. This study thus discovers a novel pathological mechanism in AD and reveals therapeutic opportunities for APOE4-driven cerebrovascular pathology. Importantly, targeting this mechanism may reduce the severe vascular side effects APOE4 carriers experience with anti-amyloid monoclonal antibody therapies.

AD has long been characterized by the presence of amyloid plaques and neurofibrillary tangles in the brain parenchyma^94^. However, cerebrovascular pathology is often one of the earliest manifestations of AD, sometimes occurring even before symptoms arise^24^. As part of disease progression, pericytes are lost along the vasculature leading to microvessel degeneration and the leakage of intravascular components into the brain parenchyma^45,95–98^. In addition to forming plaques, amyloid often accumulates along the vasculature in AD patients, leading to cerebral amyloid angiopathy^99^. Perivascular ECM accumulation is another feature of aging and AD, and several cerebrovascular ECM proteins, particularly fibronectin, are positively correlated with amyloid pathology^100–118^. Strikingly, TGF-β overexpression in mice leads to thickening of the cerebrovascular basement membrane that precedes amyloid accumulation and vascular dysfunction^53^. These findings suggest a link between TGF-β, perivascular fibrosis, and AD pathogenesis.

This study is the first to demonstrate that APOE4 directly increases TGF-β signaling along the cerebrovasculature, leading to vascular fibrosis and amyloid deposition via pericyte differentiation into myofibroblasts. Cerebrovascular degeneration is a well-characterized phenotype in APOE4 carriers that is linked to accelerated cognitive decline^25^. Independent of AD pathology, pericyte coverage is significantly reduced in APOE4 individuals and carriers of this allele are more susceptible to cerebral hemorrhages^25,91,119^. Additionally, perivascular amyloid accumulation is far more common in APOE4 carriers^91,92^. More recently, ECM dysregulation has also been implicated in APOE4-associated AD. APOE4 astrocytes and microglia upregulate matrisome pathways^10,120^ and solutes from the brain parenchyma, including amyloid, drain along the vasculature^121–124^. This suggests that perivascular accumulation of pro-aggregatory ECM proteins may drive impaired amyloid clearance^100,105^. Indeed, perivascular amyloid drainage is decreased in human APOE4-targeted replacement mice^125^. Strong evidence for a fibrotic phenotype in APOE4-mediated AD pathogenesis is a recent study that identified genetic factors protecting carriers of this allele from developing the disease^50^. They found that many protective variants are in ECM genes and that a particular loss-of-function *FN1* variant reduces the risk of developing AD by approximately 71%. Coinciding with these results, the brains of APOE4 carriers have increased perivascular fibronectin deposition compared to non-carriers^50,51^. To date, these APOE4-associated vascular pathologies – pericyte loss, amyloid deposition, and fibrosis – have all been investigated separately. However, this study uniquely identifies a TGF-β-dependent mechanism that links these phenotypes together into a single, targetable pathway. We show that inhibiting TGF-β can reverse cerebrovascular pathology in APOE4 which is, at least in part, due to reduced fibronectin accumulation. Therefore, the work presented here simultaneously explains a mechanism of AD risk and resilience and offers a novel therapeutic opportunity for APOE4 individuals.

Our study utilizes an orthogonal approach integrating single-nuclei transcriptomic and immunocytochemical analysis of the postmortem human brain with isogenic iPSC brain models and humanized APOE knock-in mice. From this rigorous approach, we discover for the first time a causal link between APOE4, TGF-β signaling, cerebrovascular fibrosis, and AD pathology, opening a path to new therapeutic opportunities in the treatment and prevention of AD in high-risk individuals.

## Resource availability

### 1. Lead Contact

Further information and requests for resources and reagents should be directed to the lead contact, Joel Blanchard (joel.blanchard@mssm.edu).

### 2. Materials availability

This study did not generate new unique reagents.

### 3. Data and code availability

All datasets utilized in this study are from publicly available sources. The code used in this study is available at https://github.com/blanchardlab/Schuldt2025. Any additional information required to reanalyze the data reported in this paper is available from the lead contact upon request.

## Methods

### Processing and subject selection of individual snRNA-seq studies

Datasets from the following studies were downloaded and processed following the original methods as a guide. All analysis was performed in R with the Seurat package^139^ unless otherwise specified.

1. Yang et al., 2022
  Raw data from hippocampal samples were downloaded from GSE163577. To pass quality control, nuclei had to meet the following criteria: (1) more than 200 features, (2) less than 5000 features, (3) less than 5% mitochondrial RNAs, and (4) less than 5% ribosomal RNAs. Doublets were filtered out using DoubletFinder^130^. The standard Seurat pipeline of *NormalizeData(), FindVariableFeatures()* (nfeatures = 2000), and *ScaleData()* was performed. The top 30 principal components (PCs) were used for UMAP generation at a resolution of 0.2. Harmony was used for batch correction^134^. Clusters were annotated using the marker genes defined in the source publication. Clusters annotated as astrocytes, endothelial cells, and mural cells were then extracted for generation of the harmonized cerebrovascular atlas.
2. Sun et al., 2023
  Processed and clustered data was downloaded from the source publication. Only individuals with APOE3/3, APOE3/4, and APOE4/4 genotypes were included. Nuclei labeled as endothelial cells, smooth muscle cells, and pericytes were extracted for construction of the cerebrovascular atlas.
3. Haney et al., 2024
  Raw data of AD samples was downloaded from GSE254205. Nuclei that passed the following quality control parameters were included for further analysis: (1) more than 500 features, (2) more than 1000 reads, (3) less than 10% mitochondrial reads, and (4) less than 10% ribosomal reads. Doublets were removed using DoubletFinder. The standard Seurat pipeline of *NormalizeData(), FindVariableFeatures()* (nfeatures = 2500), and *ScaleData()* was performed. The top 20 principal components (PCs) were used for UMAP generation at a resolution of 0.2. Harmony was used for batch correction. Clusters were annotated using the marker genes defined in the source publication. Clusters annotated as astrocytes and vascular cells were extracted.

After extracting the cerebrovascular cell types from each dataset, propensity matching for APOE4 carriers and non-carriers was performed within each dataset to minimize confounding variables and equalize the number of individuals with each genotype. Specifically, the R package MatchIt^141^ was employed to match individuals using the following parameters:

APOE~age+sex+ADstatus+totalcellcount,method=‘nearest’,ratio=1

### Cerebrovascular atlas integration and clustering

After propensity matching, data from the selected individuals were merged. The standard Seurat pipeline of *NormalizeData(), FindVariableFeatures()* (nfeatures = 2000), and *ScaleData()* was performed. Harmony was used for integration of the datasets with the following parameters:

RunHarmony(c("subject","dataset"),theta=c(2,4))

The top 15 principal components (PCs) were used for UMAP generation at a resolution of 0.5. Clusters with high expression of markers of two or more cell types were excluded from further analysis. After excluding a cluster, the Seurat pipeline and harmony integration was repeated. Ultimately, three rounds of clustering were performed and the final integrated UMAP was generated at a resolution of 0.2. Clusters were manually annotated using canonical marker genes for astrocytes, endothelial cells, pericytes, and smooth muscle cells (SMCs).

For mural cell subclustering, pericytes and smooth muscle cell clusters were extracted. *FindNeighbors(), FindClusters(),* and *RunUMAP()* were re-run with the top 15 PCs at a resolution of 0.2. Subclusters were annotated using previously described marker genes^55,56^.

### Differential abundance analysis

1. miloR
  The miloR package was implemented in R^58^. For the entire cerebrovascular atlas, a k-nearest-neighbor (KNN) graph was generated using the *buildGraph()* function with parameters k = 50 and d = 30. Neighborhoods were generated with the *makeNhoods()* function with parameters prop = 0.1, k = 50, and d = 30 followed by *calcNhoodDistance()* with d = 30. Differential abundance between APOE4 carriers and non-carriers was assessed using the *testNhoods()* function with default parameters and the following design:
Design=~age+sex+ADstatus+dataset+apoe
  For miloR differential abundance analysis of the mural cell subclusters, the same pipeline was used as above, apart from the following differences in the parameters: *buildGraph()* (k = 30, d = 30), *makeNhoods()* (k = 30, d = 30), and *calcNhoodDistance()* (d = 30) For all above functions, Harmony embeddings were used as the dimensionality reduction input.
2. DASeq
  The DAseq package was implemented in R^129^. To compute APOE4 carrier vs.non-carrier differential abundance in mural cell subclusters, Harmony embeddings were used as input to DAseq along with APOE genotype labels for each subject. A range of neighborhood sizes *(k.vector = seq(50, 500, 50))* was used to compute differential abundance (DA). Cells with DA scores above 0.7 or below −0.7 were classified as differentially abundant and visualized on UMAP coordinates. DA regions were identified using the *getDAregion()* function at a resolution of 0.05.
3. Binomial generalized linear models to estimate subject-level cell proportions
  For myofibroblast proportions, the number of cells in the SMC_2 DAseq region defined as myofibroblasts (Figure 1J) were counted for each non-AD individual. Myofibroblast proportion per individual was defined as the number of myofibroblast cells divided by the total number of SMC_2 cells. We then fit a binomial generalized linear model (GLM) with a logit link using the *glm()* function in R. The following design was used:
glm(cbind(myofibroblastcount,totalSMC2count-myofibroblastcount)~apoe+ns(age,df=3)+sex+dataset,family=binomial())
  Age was modeled with a natural cubic spline with 3 degrees of freedom to account for potential non-linear effects. Model diagnostics were assessed with the DHARMA package^142^. Model coefficients for each for the variables were extracted and expressed as odds ratios with 95% confidence intervals.
  For intermediate (*ACTA2+/CSPG4+*) cell proportions, the same analysis was performed as above except as a proportion of total mural cell count per individual.

### Differential gene expression (DGE) analysis

1. Single-cell DGE analysis of mural cell subclusters
  Single-cell differential gene expression was performed with MAST^136^ by using the Seurat *FindMarkers()* function. Pairwise comparisons between APOE4 carriers and non-carriers were performed for each mural cell subcluster. Age, sex, AD status, and dataset were included as covariates. A differentially expressed gene was defined as |logFC| > 0.25 and an adjusted p-value < 0.05.
2. Pseudobulk DGE analysis of SMC_2 cluster
  For the mural cell subclusters, gene expression counts were aggregated across cells for each subject using the *AggregateExpression()* function in Seurat, grouping by subject, APOE genotype, and mural cell type. The data was filtered to only include SMC_2 cells from individuals contributing at least 5 cells. A voom-limma^135^ pipeline was then employed to model differential gene expression between APOE4 carriers and non-carriers. The following linear model was fit for each gene:
$$Y\;=\beta_{0}+\;\beta_{1}\cdot APOE4\;+\;\beta_{2}\cdot age\;+\beta_{3}\cdot sex\;+\;\beta_{4}\cdot dataset\;+\;\beta_{5}\cdot ADstatus\;+\;\varepsilon$$
  In this model, *Y* represents the voom-transformed expression value of gene *i* in subject *j*; *APOE4* is an indicator variable for APOE4 genotype (with APOE3 as the reference); *age* , *sex* , *dataset* , and *ADstatus* represent the subject-level covariates for age, sex, dataset, and AD status, respectively; *β*_0_ is the model intercept; *β*_*1*_*–β*_*5*_ are the estimated coefficients for each predictor; and ε is the residual error term. For each gene, statistics were calculated using the *eBayes()* function.
3. Myofibroblast marker genes
  DGE was performed with MAST by using the Seurat *FindMarkers()* function. Within the SMC_2 subcluster, a pairwise comparison was performed between the cells in the APOE4-enriched DAseq region (Figure 1J) and the remaining SMC_2 cells. Age, sex, AD status, and dataset were included as covariates. A myofibroblast marker gene was defined as logFC > 0.25 and an adjusted p-value < 0.05.

### Pathway analysis

Gene set enrichment analysis (GSEA) was performed using the fgsea R package^131^. The REACTOME database was input via the msigdbr R package^138^. Following APOE4 carrier vs. non-carrier differential gene expression analysis via MAST or voom-limma (see differential gene expression analysis methods section), genes were ranked according to the following formula:

$$ranking\;score\;=\;sign(avg\_{log}_{2}FC)\;\times \;-{log}_{10}(p-value)$$

Genes were then sorted in decreasing order and input into the fgsea package. The enrichment score, normalized enrichment score, p-values, and FDR-adjusted p-values were computed for each pathway. Significant pathways were defined as an adjusted p-value < 0.05. Redundant pathways were merged to enhance readability.

For Gene Ontology (GO) enrichment analysis, differentially expressed genes were input into the ClusterProfiler R package^127^ using the enrichGO() function with *ont = “ALL”.* Redundant pathways were merged with the *simplify()* function. Significant pathways were defined as an adjusted p-value < 0.05.

### Unbiased cell type annotation

Cell type annotation was performed with the CellMarker_Augmented_2021 database via the enrichR package^77,143^. APOE4-enriched genes from the pseudobulk SMC_2 DGE analysis (logFC > 0.25, p < 0.05, see DGE analysis methods) or the myofibroblast marker genes (see DGE analysis methods) were input into enrichR with default parameters. Only cell types with at least 10 marker genes and 3 hits were included. Redundant cell types were merged to enhance readability.

### Seurat module scores

1. Cellular contraction, extracellular matrix (ECM), and co-expression scores in mural cells
  Module scores were calculated for each cell using the *AddModuleScore()* function in Seurat. The following genes were used for each module:
Contraction:ACTA2,TAGLN,ITGA1,ITGA2,ITGA8,CDH11,PALLD,SORBS1,LMOD2,TPM2
ECM:COL4A1,COL6A2,COL1A1,COL3A1,COL1A2,COL5A2,COL14A1,FN1,FBN1,DCN
Co-expression:Minimumofthetwoscoresforeachcell
  For comparison of module scores in APOE4 carriers vs. non-carriers, the data was filtered to only include SMC_2 cells from individuals contributing at least 5 cells. The module scores were then averaged across all cells per individual for subject-level comparisons between APOE genotypes.
2. TGF-β signaling in pericytes
  The Pericyte_1 and Pericyte_2 mural cell subclusters were extracted and a module score was calculated for each cell using the *AddModuleScore()* function in Seurat. The following genes were used for the module:
TGF-βsignaling:TGFβR1,TGFβR2,TGFβ1,TGFβ2
  For comparison of TGF-β gene expression in APOE4 carriers vs. non-carriers, the module score was then averaged across all cells per individual for subject-level comparisons between APOE genotypes.

### Myofibroblast gene activity scores

Gene set variation analysis was performed using the GSVA R package^133^.

1. Human cerebrovascular atlas
  The data was filtered to only include SMC_2 cells from individuals contributing at least 5 cells. Gene expression counts were aggregated across cells for each subject using the *AggregateExpression()* function in Seurat, grouping by subject and APOE genotype. Myofibroblast marker genes were downloaded from a previous publication (cluster 11)^80^ and GSVA scores were assigned to each subject using the top 10 highly expressed myofibroblast marker genes in our dataset.
2. APOE-TR mice
  The processed and normalized 4-month-old mouse bulk RNA-seq data was downloaded from Synapse (syn26561824)^81^. GSVA scores were calculated for each sample using the myofibroblast marker genes from our cerebrovascular atlas (see myofibroblast marker genes in the DGE analysis methods section). The human genes were converted to their mouse orthologs prior to GSVA input.

### Pseudotime analysis

1. Lineage assignment

Lineages were generated using the slingshot R package^140^. The UMAP of mural cell subclusters was input into slingshot with the Pericyte_2 cluster assigned as the start cluster.

- Lineage bias

For the lineages predicted by slingshot, curve weights were assigned to each cell. The R package condiments^128^ was then used to test for differential lineage assignment between APOE genotypes. Specifically, the *differentationTest()* function was performed for this anlaysis.

- Generalized additive models (GAMs)

Three sets of module scores were assigned to mural cells using the *AddModuleScore()* function in Seurat with the following genes:

Pericytesignaturescore:PDGFRB,ANPEP,CSPG4,KCNJ8,SLC20A2,SLC6A1

Myofibroblastsignaturescore:theco-expressionscoredefinedintheSeuratmodulescoresmethodssection

TGF-βsignalingsignaturescore:thehallmarkTGF-βsignalinggeneset

After assigning scores, separate GAMs with slingshot-derived lineage weights were fit for each module score with a smoothing parameter of *k = 7* using the mgcv R package^137^. To compare scores between lineages, the differences in areas under the curve (Δ-AUC: pericyte-to-myofibroblast – pericyte-to-pericyte lineage) was calculated for each GAM. Permutation testing (n = 1000) was then used to generate a null distribution of Δ-AUC values. A two-sided p-value was calculated as the proportion of permuted Δ-AUC values exceeding the observed Δ-AUC in magnitude.

### NicheNet

The NicheNet R package was utilized for this analysis^93^. A sender-focused approach was performed. The DAseq APOE4-enriched myofibroblast region (Figure 1J) was assigned as the receiver cell type. The astrocyte, endothelial, pericyte_1, pericyte_2, SMC_1, SMC_2, and myofibroblast clusters were assigned as the sender cell types. Genes expressed in at least 5% of receivers or senders were used in the analysis, and the differential genes input into NicheNet for ligand prediction were the myofibroblast marker genes defined in the DGE analysis methods section.

### Association of mural cell *FN1* expression with AD phenotypes

Mural cells from the Haney (2024) dataset^11^ were extracted from the cerebrovascular atlas and *FN1* expression was averaged per individual. Mural cell *FN1* expression was then correlated with age of AD diagnosis. A global cerebral pathology score was assigned to each individual by averaging the following metadata columns reported in the original dataset: *sum_lb_density, PlaqueTotal, TangleTotal, infarct_cerebral_total_volume.* Scores were then dichotomized into high (top 25%) and low (bottom 75%) pathology groups to compare mural cell *FN1* expression between them.

### Bulk RNA-seq analysis of iPSC-derived mural cells (iMCs)

FPKM values were analyzed from a previous study^47^. After log-transforming the FPKM values, differential expression analysis between APOE3/3 and APOE4/4 iMCs was performed using the standard limma pipeline. Array weights were estimated using the *arrayWeights()* function and statistics were calculated for each gene using the *eBayes()* function with *trend = TRUE* and *robust = TRUE.* A differentially expressed gene was defined as |logFC| > 0.25 and an adjusted p-value < 0.05. Differentially expressed genes were input into ClusterProfiler for GO pathway analysis. Significant pathways were defined as an adjusted p-value < 0.05.

For CIBERSORTx^85^ deconvolution, the web-based application was used. Mural cell subclusters from the cerebrovascular atlas were used as the reference. Specifically, 10,000 mural cells were randomly downsampled from the atlas, and their gene counts were extracted. A signature matrix was generated using standard parameters, with a sampling ratio of 0.5 and inclusion of genes expressed in at least 25% of cells. Cell type proportions were then estimated from the bulk RNA-seq gene expression data using default CIBERSORTx settings. For downstream analysis, cell type proportions in APOE4/4 samples were expressed as log fold changes relative to the mean proportions in APOE3/3 samples.

### Human iPSC cultures

Pre-coated Geltrex ^™^ Matrix plates were seeded with iPSCs. iPSC colonies were then grown in StemFlex ^™^ medium until 60–70% confluency. Subsequently, maintenance iPSCs were passaged using 0.5 mM EDTA to gently lift colonies. iPSCs for differentiation were harvested with Accutase ^™^ cell detachment solution for 5–10 minutes at 37C and differentiated as single cells according to the specified differentiated protocol.

### Differentiation of human iPSCs into neurons

Protocol for neuron differentiation was adapted from Zhang et al.^144^ and Lam et al.^145^. In brief, Lipofectamine^™^ Stem Transfection Reagent was used to transfect iPSCs with PiggyBac plasmids to confer doxycycline-inducible expression of the Neurogenin-2 gene (*NGN2*, Addgene Plasmid #209077). In short, dissociated iPSCs were seeded on day zero at ~104,000 cells/cm^2^ onto plates coated with Geltrex^™^. The seeded cells then received StemFlex^™^ media containing 10µM Y27632 and 5µg/mL doxycycline supplements. On day 1, culture medium was replaced with Neurobasal N2B27 medium (Neurobasal, 1x B-27, 1x N-2, 1x MEM-NEAA, 1x GlutaMax, 1% penicillin-streptomycin) containing 10µM SB431542, 100nM LDN, 5µg/mL doxycycline, and 5µg/mL puromycin supplements. On days 3–6, daily medium changes were performed with Neurobasal N2B27 media containing 1µg/mL puromycin and 5µg/mL doxycycline supplements. On day 6, cells were treated with 0.5 µM Ara-C. On day 7, cells were lifted with accutase for integration into miBrains.

### Differentiation of human iPSCs into astrocytes

Previously published iPSC-derived NPC (Chambers et al.)^146^ and astrocyte (TCW et al.)^10^ differentiation protocols were followed to generate astrocytes. In summary, dissociated iPSCs were seeded onto Geltrex ^™^ - coated plates at a cell density of 100,000 cells/cm^2^ in pre-warmed StemFlex ^™^ media containing 10µM Y27632 supplement. Cells received StemFlex ^™^ every other day until cells reached 95% confluence. Once confluent, cells received NPC medium (1:1 DMEM/F12: Neurobasal Medium, 1x N-2 Supplement, 1x B-27 Serum-Free supplement, 1x GlutaMAX Supplement, 1x MEM-NEAA, 1% penicillin-streptomycin) containing 10µM SB43152 and 100nM LDN193189 supplements (day 0). From days 1 to 9, cells received daily media changes with the same media as day 0. On day 10, cells were split with Accutase and seeded onto plates coated with Geltrex ^™^ and were grown in NPC media supplemented with 10 µM Y27632 and 20ng/mL bFGF. From days 11 through 13, cells received NPC media supplemented 20 ng/mL bFGF. On day 14, cells were split with Accutase and replated onto Geltrex ^™^ -coated plates and received NPC media supplemented with 10µM Y27632 and 20ng/mL bFGF. From day 15 onward, cells received fresh Astrocyte Medium (AM, ScienCell) every 2–3 days and were passaged using Accutase upon reaching 90% confluence. From this point on, NPCs were differentiated into astrocytes over a 30-day time course. NPCs and differentiated astrocytes were then cryopreserved in freezing medium, which consisted of 90% knockout serum replacement (KSR) and 10% dimethyl sulfoxide (DMSO).

### Differentiation of human iPSC into brain microvascular endothelial cells

Brain endothelial cells were differentiated according to adapted protocols outlined in Blanchard et al.^47^, Qian et al.^147^, and Wang et al.^148^. In summary, doxycycline-inducible expression of the ETS variant transcription factor 2 (*ETV2*, Addgene Plasmid #168805) was conferred in iPSCs by transfecting iPSCs with a PiggyBac plasmid. Inducible *ETV2*-iPSCs were cultured until 60–70% confluence before they were dissociated with Accutase and seeded at 20,800 cells/cm^2^ onto plates coated in Geltrex ^™^ that contained StemFlex^™^ supplemented with 10µM Y27632 on day 0. On day 1, culture medium was replaced with DeSR1 medium (DMEM/F12 with GlutaMAX, 1x MEM-NEAA, 1x penicillin-streptomycin) containing 10 ng/mL BMP4, 6µM CHIR99021, and 5µg/mL doxycycline supplements. On days 5 and 7, cell media was changed and replaced with hECSR medium (Human Endothelial Serum-free Medium, Gibco; 1x MEM-NEAA; 1 x B-27, 1% penicillin-streptomycin) that was supplemented with 50 ng/mL VEGF-A, 2µM Forskolin, and 5µg/mL doxycycline. On day 8, cells were lifted with Accutase and re-plated onto fresh plates coated with Geltrex ^™^. Cells received hECSR media that was supplemented with 50 ng/mL VEGF-A and 5 µg/mL doxycycline. In the week following day 8, the supplemented day 8 medium was given to cells every 2–3 days to maintain cells until they were ready for tissue assembly in miBrains.

### Differentiation of human iPSCs into mural cells

Mural cell differentiation protocol was based on published protocol from Patsch et al.^149^. Dissociated iPSCs were seeded at 37,000 to 40,000 cells/cm^2^ onto plates coated with Geltrex^™^ and grown in StemFlex ^™^ containing 10µM Y27632 supplement on day 0. On day 1, StemFlex ^™^ medium was replaced with N2B27 medium (1:1 DMEM/F12, Neurobasal media, 1x B-27, 1x N-2, 1x MEM-NEAA, 1x GlutaMAX, and 1% penicillin-streptomycin) containing 25 ng/mL BMP4 and 8µM CHIR99021 supplements. On days 3 and 4, cultures received a media change with N2B27 media supplemented with 10 ng/mL Activin A and 10 ng/mL PDGF-BB. On day 5, pericytes were lifted with accutase, plated at 35,000 cells/cm^2^ onto plates coated in 0.1% gelatin, and grown in N2B27 containing 10 ng/mL PDGF-BB supplement. Cells received media changes with day 5 media every 2–3 days until confluent, at which point they were either frozen in freezing medium (90% KSR/10%DMSO) in liquid nitrogen tanks or split onto 0.1% coated gelatin plates in N2B27 media and maintained until ready for miBrain tissue assembly. For monoculture experiments, cells were plated onto 96-well µClear plastic-bottom plates (Greiner) coated with 0.1% gelatin at a density of 2500–5000 cells per well, and fixed 96 h post-plating unless specified otherwise.

### Differentiation of human iPSCs into oligodendrocyte progenitor cells

At nearly 100% confluence, iPSCs in mTeSR1 with 10 µM Y27632 were plated on Matrigel (day-1). Every day until day 7, 2 mL of neural induction media (DMEM/F12 with 1x NEAA, GlutaMAX, 2-mercaptoethanol, and penicillin-streptomycin), with 10 μM SB431542, 250 nM LDN193189, and 100 nM RA (freshly added per use) was added. From day 8 through day 11, media was replaced daily with N2 medium (DMEM/F12 with 1x NEAA, GlutaMAX, 2-mercaptoethanol, and penicillin-streptomycin), along with 1x N2 and 100 nM RA and 1uM SAG (added fresh daily). On day 12, a cell scraper lifted the cells, which were gently transferred to a 6-well ultra-low attachment plate with 3 mL of OPC-N2B27 medium (DMEM/F12 with 1x NEAA, GlutaMAX, 2-mercaptoethanol, penicillin-streptomycin, 1x N2, 1x B27 without Vit A, 25 µg/=L insulin, and freshly added 100 nM RA and 1 µM SAG). About two-thirds of the OPC-NB27 media was changed every two days until day 20, without disturbance of the cell aggregates. From day 21 until day 30, PDGF medium (DMEM/F12 with 1x NEAA, GlutaMAX, 2-mercaptoethanol and penicillin-streptomycin, 1x N2, 1X B27 without Vit A, 25 ug/ml insulin, 100 ng/ml biotin, 1 µM cAMP, 5 ng/ml HGF, 10 ng/ml IGF-1, 10ng/ml NT3, and 10 ng/ml PDGFaa) was added every other day. On day 30, aggregates measuring 300–800 μm in size were picked with a p200 pipette and plated on PLO/laminin-coated 6-well adherent plates. Cells were plated at a density of 20 cells per well in PDGF medium. Until day 75, two-thirds of the PDGF media was changed every other day. PDGF media was then changed every 2–3 days, and the differentiated OPCs were enriched through FACS with a lineage-specific marker. OPC differentiation followed the protocol from Douvaras et al.^150^.

### Differentiation of human iPSCs into microglia

To generate microglia, iPSCs were first differentiated into hematopoietic progenitor cells (HPCs) using STEMdiff^™^ Hematopoietic Kit (Catalog #05310). On days 12, 14, and 16 of the differentiation, floating HPCs were collected from media before freezing in CellBanker (AMSBIO). Following week 2 of miBrain assembly, HPCs were seeded into the existing tissue (25000 / 10 μL miBrain). miBrains were then maintained in miBrain media supplemented with 100ng/mL IL34 and 25ng/mL M-CSF. Microglia differentiation protocol was adapted from previous studies^151,152^.

### 3D Tissue Assembly for miBrains

Neurons, endothelial cells, mural cells, and OPCs were lifted with Accutase, and astrocytes in TrypLE Select. Cells were then resuspended in their respective media, counted, and resuspended at 1 x 10^6^ cells/mL. To assemble miBrains, a 15 mL Falcon tube for each miBrain condition was prepared to receive approximately 5 x 10^6^ neurons, 5 x 10^6^ endothelial cells, 1 x 10^6^ astrocytes, 1 x 10^6^ OPCs, and 1 x 10^6^ pericytes per 1 mL. Each tube of pooled cells was spun down for five minutes at RT at 200 x g. After carefully aspirating as not to disturb the cell pellet, each resulting pellet was placed on ice before resuspension in 1 mL Geltrex^™^ ,10 µM Y27632 and 5 µg/mL doxycycline. Resuspension technique took care to avoid air bubbles, and cell pellets were kept on ice to prevent premature Geltrex polymerization and ensure successful seeding. 10 µL of encapsulated cell suspensions were then plated per well of a 96-well µClear plastic-bottom plate (Greiner). To allow the Geltrex^™^ to polymerize, seeded plates were transferred into a 37 °C 95%/5% Air/CO2 incubator for 30 minutes. After polymerization, miBrain cultures were completely submerged in miBrain week-1 medium (Human Endothelial Serum-free Medium, 1x Pen/Strep, 1X MEM-NEAA, 1X CD Lipids, 1x Astrocyte Growth Supplement (ScienCell), 1x B27 Supplement, 10ug/mL Insulin, 1µM cAMP-dibutyl, 50µg/mL Ascorbic acid, 10ng/mL NT3, 10ng/mL IGF, 100ng/mL Biotin, 60 ng/mL T3, 50 ng/mL VEGF, 1µM SAG, 5 µg/mL doxycycline). Each well received 200 μL of media. Every 2–3 days, a half-media change was performed until day 8, before changing the media to miBrain week-2 medium (Human Endothelial Serum-free Medium, 1x Pen/Strep, 1X MEM-NEAA, 1X CD Lipids, 1x Astrocyte Growth Supplement (ScienCell), 1x B27 Supplement, 10ug/mL Insulin, 1µM cAMP-dibutyl, 50µg/mL Ascorbic acid, 10ng/mL NT3, 10ng/mL IGF, 100ng/mL Biotin, 60 ng/mL T3, 5 µg/mL doxycycline). After 4 weeks, miBrain cultures were considered ready for downstream assays.

### 3D tissue assembly for iPSC-derived endothelial cell monocultures

This protocol is the same as the miBrain protocol with the following differences. Each 10 µL Geltrex droplet contained 120,000 of only endothelial cells. Cultures were maintained in hECSR media supplemented with 50 ng/mL VEGF and 5 µg/mL doxycycline for two weeks until fixation (see immunofluorescence methods section).

### Transduction of iPSC-derived mural cells with lentiviral shRNAs

FN1 and scramble MISSION lentiviral shRNAs were purchased from Sigma Aldrich. Lentivirus was generated in HEK293T cells following standard protocols. iPSC-derived mural cells were then transduced at a 1:40 dilution in mural cell culture media. Media was replaced 24 hours after transduction, and puromycin selection was initiated 72 hours post-transduction. Puromycin selection was maintained until cells were integrated into miBrains or collected for qRT-PCR validation of *FN1* knockdown.

### Amyloid and fibronectin treatments

3D iPSC-derived endothelial cell monocultures were generated as previously described. For the amyloid treatment group, 20 nM of both amyloid-β 1–40 and 1–42 were incubated in cell culture media for 1 hour at RT. For the amyloid and fibronectin treatment group, 20 nM of both amyloid-β 1–40 and 1–42 were incubated with 50 ng/mL fibronectin for 1 h in cell culture media at RT. Cells were treated with these mixtures for 96 h before fixing (see immunofluorescence methods section).

### TGF-β ligand treatments

iPSC-derived mural cells were seeded onto 96-well plates as previously described. 24 h post-plating, cells were treated with 50 ng/mL TGFβ1 for 96 hours followed by fixation (see immunofluorescence methods section).

### TGF-β inhibitor treatments

For 2D treatments, iPSC-derived mural cells were seeded onto 96-well plates as previously described. 24 hours post-plating, cells were treated with 10 µM SB431542 for 96 hours followed by fixation. For miBrains, treatments were either initiated at 3–4 days post-plating and maintained for 4 weeks until fixation or initiated at 2 weeks post-plating and maintained for the remaining 2 weeks until fixation, as specified in the figure legends. The concentrations used for miBrains were 10 µM SB431542 and 10 µM Galunisertib.

### Immunofluorescence of cell cultures

2D cultures were fixed in 4% paraformaldehyde for 15 minutes at room temperature, rinsed with PBS and permeabilized in 0.1% Triton-X100 in PBS for 10 minutes. Cultures were then blocked in 5% normal donkey serum in PBS for 2 h. Primary antibodies diluted 1:500 in blocking solution were added to cultures and incubated overnight at 4°C, before 3 consecutive rinses with PBS for 10, 20, and 30 minutes. Cultures were then incubated for 2h at room temperature with secondary antibodies diluted 1:1000 in blocking solution. After rinsing once with PBS for 10 min, cultures were incubated for 20 min in Hoechst 33258 (Sigma-Aldrich, Cat. no. 94403) diluted at 1:1000 PBS. Cultures were then rinsed once more with PBS for 30 min. The PBS was then replaced prior to imaging. See corresponding image acquisition and quantification section.

3D cultures and miBrains were incubated overnight at 4°C in blocking solution (0.3% Triton-X100, 5% normal donkey serum, in PBS). Primary antibodies, diluted 1:500 in blocking solution, were added the following day and left for 2–3 nights at 4°C. Cultures received 5 x 30 min rinses with 0.3% Triton-X100 PBS before adding secondary antibodies and Hoechst. Both were diluted at 1:1000 in blocking solution and cultures were left to incubate at 4°C for 1–3 nights. Cultures were rinsed for 5 x 30 min with 0.3% Triton-X100 PBS, before rinsing once more and leaving cells in regular PBS for image acquisition. See corresponding image acquisition and quantification section.

### Mouse models

All experiments were performed in accordance with the Guide for the Care and Use of Laboratory Animals and were approved by the Institutional Animal Care and Use Committee at the Icahn School of Medicine at Mount Sinai (ISMMS). For all mouse experiments, C57BL/6J background mice with their endogenous murine *Apoe* replaced with human APOE3/3 or APOE4/4 were used. Aged, retired breeder APOE-TR mice were purchased from Jackson Laboratories (APOE3/3-TR strain number: 029018; APOE4/4-TR strain number: 027894). Ages ranged from 8–10 months and were females. Mice were housed in the ISMMS vivarium under standard conditions until experimentation.

### Immunofluorescence of mouse brain tissue

Mice were first anaesthetized via gaseous isoflurane exposure, followed by cardiac perfusion with ice-cold PBS and subsequently 4% paraformaldehyde (PFA). The brains were dissected out and post-fixed in 4% PFA at 4C overnight. Fixed brains were placed in 30% sucrose in PBS until the tissue had sunk and were then embedded in OCT. Slices were cut at a thickness of 40 µm using a Leica cryostat and stored at −20C in cryoprotectant solution until staining.

For free-floating immunofluorescence, sections were carefully transferred into a 24-well plate with fresh PBS and rinsed two more times. Slices were then blocked in a buffer containing 0.3% Triton X-100 and 5% normal donkey serum in PBS overnight at 4˚ C. Primary antibodies diluted in blocking buffer were added the following day for 2–3 nights at 4˚ C. Sections were washed 5 x 30 min in PBS, and secondary antibodies diluted in blocking buffer were added for 2 hours at RT. Slices were then washed 5 x 30 min in PBS and mounted on glass slides for imaging. For slices stained with lectin, lectin was diluted 1:200 in PBS as part of the first wash and left for 1 h. Hoechst was added 1:1000 in PBS as part of the second wash and left for 30 min. All images were acquired in the corpus callosum and in approximately the same locations of each slice to minimize biasing the results due to differences in anatomical regions (see image acquisition and quantification methods section).

### Image acquisition and quantification

Images were acquired using a Nikon AX R confocal microscope. The same parameters were used for each image within an experiment, and the experimenter was blinded to the channels of interest or experimental condition during imaging. Images were analyzed using scripts from the Nikon AX R built-in quantification software, CellProfiler^126^, or FIJI/ImageJ^132^. Statistical analysis was performed with GraphPad Prism or R and the tests used are specified in each figure caption.

## Extended Data

**Extended Data Figure 1.**
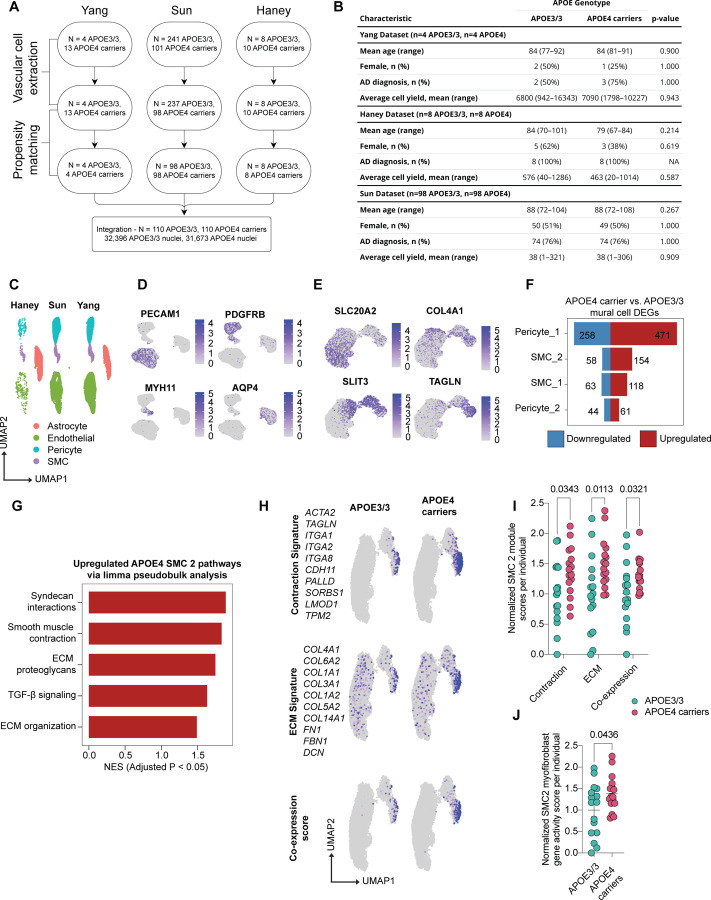
Metadata, quality control, and validation of single-nuclei analyses. **A.** Flowchart describing the generation of the final integrated cerebrovascular atlas used for downstream analyses. Astrocytes, endothelial cells, pericytes, and smooth muscle cells were extracted from each individual. Within each dataset, propensity matching based on age, sex, Alzheimer’s disease (AD) status, and vascular nuclei contribution was performed using the MatchIt package. **B.** Quality control of propensity matching. Unpaired two-sample Student’s t-tests were used to compare age and average vascular cell yield. Chi-squared tests were used to compare age and AD status for the Sun dataset. These two variables were compared using Fisher’s Exact Test in the Yang and Haney datasets. **C.** UMAP of each dataset after integration. **D.** Annotation of blood brain barrier cell types (*PECAM1* = endothelial cells, *PDGFRB* = pericytes, *MYH11* = smooth muscle cells, *AQP4* = astrocytes). **E.** Annotation of mural cell subclusters (*SLC20A2* = Pericyte_1, *COL4A1* = Pericyte_2, *SLIT3* = SMC_1, *TAGLN* = SMC_2). **F.** Number of differentially expressed genes between APOE4 carriers and APOE3/3 mural cell subclusters. Analysis was performed using the MAST package, and differential expression was defined as adjusted p-value < 0.05 and |logFC| > 0.25. **G.** Validation of upregulated pathways in SMC_2 cells from APOE4 carriers using a pseudobulking approach (see Methods). After statistical analysis with the limma R package, genes were ranked according to sign(logFC) * −log10(P-value) and input into the fgsea package using the REACTOME database. Significantly upregulated pathways (NES > 0, FDR < 0.05) related to the extracellular matrix and contraction were then annotated, aligning with the results in Figure 1H. **H.** Feature map of mural cells annotated for expression of contraction or extracellular matrix (ECM) gene programs. Module scores were calculated using Seurat’s *AddModuleScore()* function based on the specified gene sets. A co-expression score was defined for each cell as the minimum of its contraction and ECM scores. **I.** Comparing SMC_2 contraction, ECM, and co-expression signature scores between APOE4 carriers and APOE3/3 individuals. Scores for SMC_2 cells calculated in H. were averaged per individual. Individuals contributing less than 5 cells were excluded from the analysis. Data points represent individuals (N = 16 per genotype). Bars are group means ± SEM. P-values were calculated using unpaired two-sample Student’s t-tests. **J.** Myofibroblast gene activity in SMC_2 cells by APOE genotype. SMC 2 gene expression was aggregated per individual. Individuals contributing less than 5 cells were excluded from the analysis. Gene set variation analysis (GSVA) was then performed using the GSVA R package. GSVA scores were assigned to each individual using previously described myofibroblast marker genes (see Methods). Data points represent individuals (N = 16 per genotype). Bars are group means ± SEM. P-values were calculated using unpaired two-sample Student’s t-tests.

**Extended Data Figure 2.**
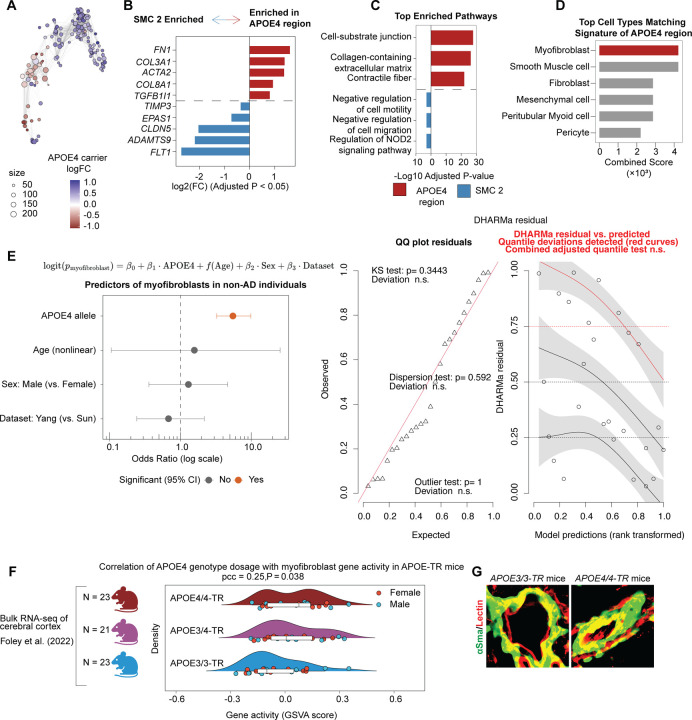
Additional validation of single-nuclei analyses. **A.** UMAP of significantly differentially abundant (DA) cell neighborhoods in mural cells of APOE4 carriers vs. non-carriers using the miloR package (spatial FDR < 0.05, positive logFC indicates enrichment in APOE4 carriers). **B.** Representative marker genes of APOE4-enriched SMC_2 cells and non-enriched SMC_2 cells identified via DAseq in Figure 1J. The MAST package was utilized to identify differentially expressed genes (|logFC| > 0.25, adjusted p < 0.05) between these two subregions. **C.** The top three enriched GO pathways in APOE4-enriched cells compared to the remaining SMC_2 cells. The differentially expressed genes identified in B. were input into ClusterProfiler. **D.** Top cell type annotations of APOE4-enriched SMC_2 cells. The differentially expressed genes upregulated in APOE4 SMC_2 cells from B. were input into EnrichR with the CellMarker database. Redundant cell types were merged to enhance readability. **E.** Binomial generalized linear model identifying predictors of myofibroblasts in non-AD individuals. The proportion of myofibroblasts relative to total SMC_2 cell count per non-AD individual (P_myofibroblast_) was modeled using a binomial generalized linear model with a logit link. Predictor variables included APOE genotype, age (modeled using a natural spline with 3 degrees of freedom), sex, and dataset. Model diagnostics were evaluated using the DHARMa package. Odds ratios (points) and 95% confidence intervals (error bars) are shown for each predictor. **F.** Correlation of APOE4 dosage with myofibroblast gene activity in APOE-TR mice. A processed bulk RNA sequencing dataset from the cerebral cortices of 4-month-old APOE3/3, APOE3/4, and APOE4/4-TR was downloaded from the source publication. Gene set variation analysis (GSVA) was performed using the GSVA R package. GSVA scores were assigned to each sample using the APOE4 myofibroblast marker genes identified in Extended Data Figure 1B. Pearson correlation analysis was subsequently performed between GSVA scores and APOE4 genotype dosage, encoded as 0 for APOE3/3, 1 for APOE3/4, and 2 for APOE4/4. **G.** Representative images of large cerebral vessels stained for lectin and αSma in age- and sex-matched APOE3/3 and APOE4/4-TR mice.

**Extended Data Figure 3.**
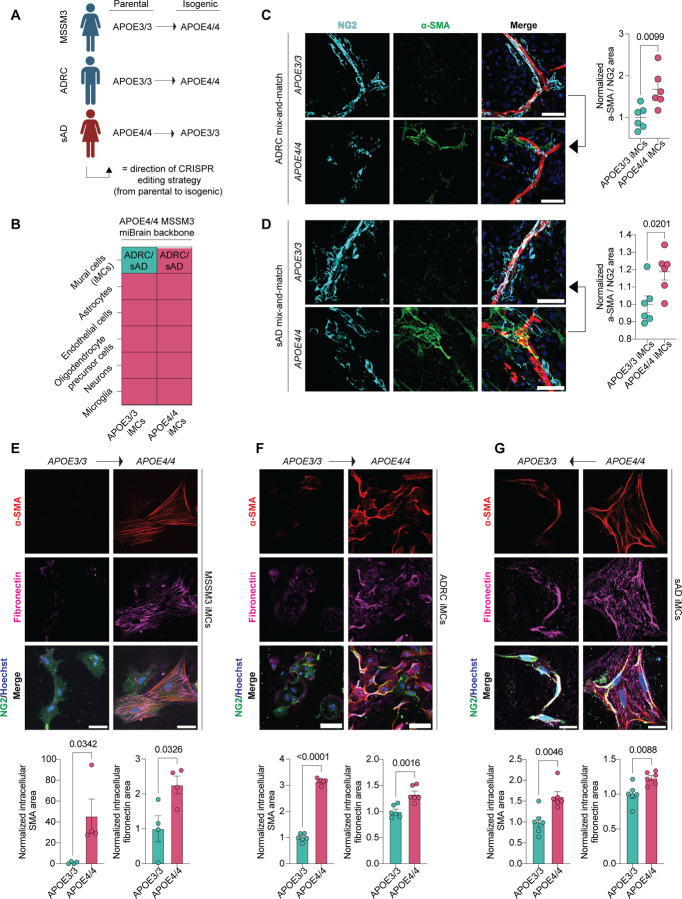
Validation of APOE4-induced myofibroblast accumulation in other iPSC lines. **A.** Characterization of iPSC lines utilized in this study. **B.** Schematic of mix-and-match miBrain experiments performed with additional isogenic pairs of iPSC-derived mural cells (iMCs). **C-D.** miBrains were immunostained for αSMA, NG2, and PECAM. Scale bar, 50 µm. The αSMA and NG2 area ratio was quantified and expressed relative to APOE3/3. Data points represent mean values from individual miBrains (n = 6 images per miBrain, n = 6 miBrains per genotype). Bars are mean group values ± SEM. P-values were calculated using unpaired two-sample Student’s t-tests. **E-G.** Representative images of isogenic iMCs in monoculture stained for NG2, αSMA, and fibronectin. MSSM3 scale bar, 50 µm. ADRC scale bar, 100 µm. SAD scale bar, 25 µm. Intracellular a-SMA and fibronectin areas were quantified as the positively stained regions within the NG2 mask, normalized to the total NG2 area, and expressed relative to APOE3/3 levels. Data points represent mean values from individual wells (n = 6 images per well, n = 4–6 wells per genotype). Bars are mean group values ± SEM. P-values were calculated using unpaired two-sample Student’s t-tests.

**Extended Data Figure 4.**
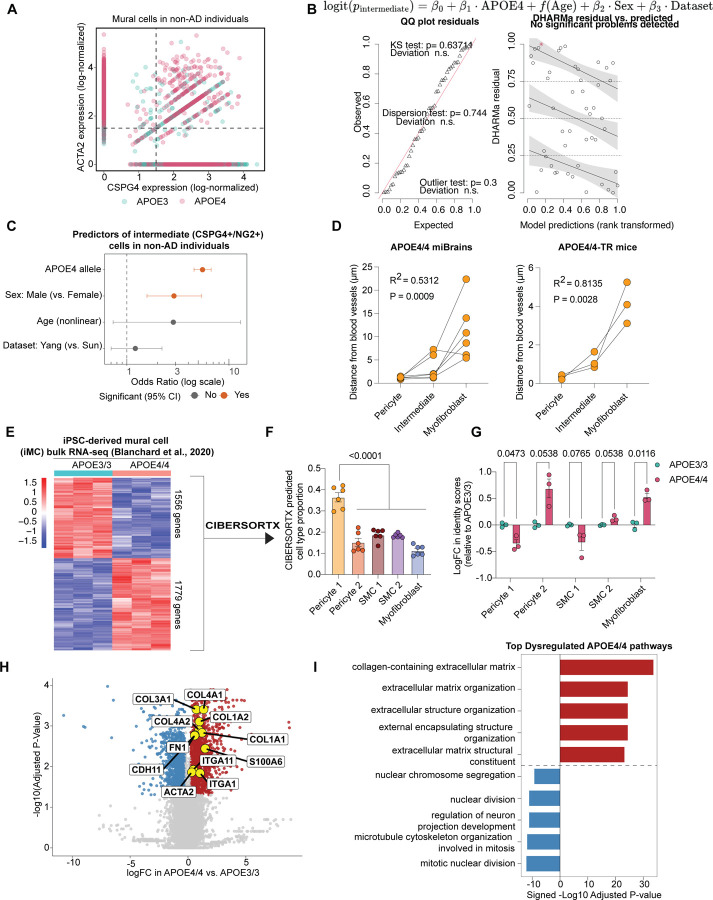
Additional computational validation of the APOE4 pericyte-to-myofibroblast transition. **A.** Cell plot used for density graph generation in Figure 2G. **B.** Binomial generalized linear model identifying predictors of intermediate cells (*ACTA2*+/*CSPG4*+) in non-AD individuals. The proportion of intermediate cells relative to total mural cell count per non-AD individual (P_intermediate_) was modeled using a binomial generalized linear model with a logit link. Predictor variables included APOE genotype, age (modeled using a natural spline with 3 degrees of freedom), sex, and dataset. Model diagnostics were evaluated using the DHARMa package. **C.** Odds ratios (points) and 95% confidence intervals (error bars) are shown for each predictor. **D.** Blood vessel distance measurements for pericytes, intermediate cells, and myofibroblasts in APOE4/4 miBrains and APOE4/4-TR mice. Cells were classified as described in Figure 2H. The *morph()* function in CellProfiler was used to calculate the distance of each pixel from the nearest PECAM+/lectin+ vascular region. For each classified cell, vascular distance was defined as the median pixel distance of all pixels within that cell. Distances were then averaged per miBrain or per mouse. Data points represent mean values from individual APOE4/4 miBrains or APOE4/4-TR mice (n = 6 miBrains, n = 3 mice). P-values were calculated using a repeated measures one-way ANOVA with a post-hoc test for a linear trend. **E.** CIBERSORTx deconvolution of bulk RNA-seq data from iPSC-derived mural cells (iMCs) using the mural cell subclusters from the single-nuclei cerebrovascular atlas as the reference. **F.** Predicted relative abundance of mural cell subclusters in iMC bulk RNA-seq data via CIBERSORTx deconvolution. **G.** Relative abundance of mural cell subclusters stratified by APOE genotype. Bars are mean logFC values ± SEM in APOE4/4 identity scores relative to APOE3/3. Data points represent individual bulk RNA-sequencing replicates (n = 3 per genotype). P-values were calculated using multiple unpaired two-sample Student’s t-tests with the Holm-Sidak correction for multiple comparisons. **H.** Representative myofibroblast marker genes upregulated in APOE4/4 iMCs (|logFC| > 0.25, adjusted p < 0.05 calculated from limma package). **I.** Top dysregulated GO pathways in APOE4/4 vs. APOE4/4 iMCs. Differentially expressed genes from H. were input into ClusterProfiler.

**Extended Data Figure 5.**
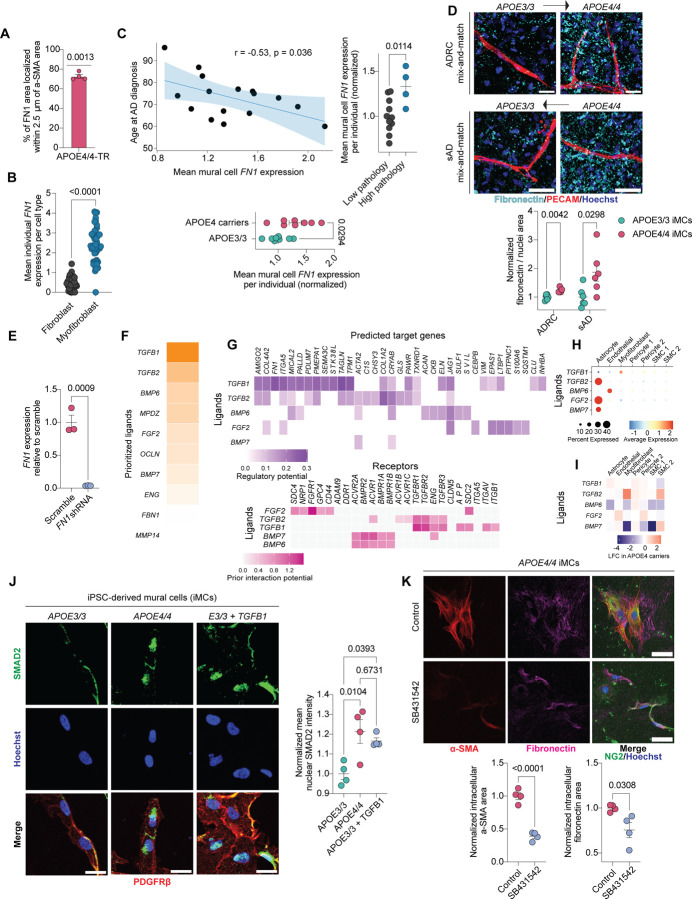
Additional experiments and computational analysis of fibronectin and TGF-B signaling in the APOE4 pericyte-to-myofibroblast transition. **A.** Fibronectin deposition near αSMA staining in APOE4/4-TR mice. The percentage of fibronectin area within 2.5 µm of αSMA-positive area relative to the total fibronectin-positive area was quantified. Data points represent mean values from individual mice (n = 6 images per mouse, n = 4 mice). Bars are mean group values ± SEM. P-values were calculated using a one-sample t-test comparing the APOE4/4-TR mean to 50%. **B.** Comparison of *FN1* gene expression in fibroblasts versus myofibroblasts. Gene expression was aggregated per individual. Data points represent individuals. Bars are group means ± SEM. P-values were calculated using a paired two-sample Student’s t-test. **C.** Association of *FN1* expression in mural cells with AD phenotypes. Mural cell *FN1* expression was aggregated per individual from the Haney dataset. A global pathology score was calculated for each individual by averaging their amyloid plaque, neurofibrillary tangle, infarct, and Lewy body scores reported in the source metadata. These pathology scores were classified as low (bottom 75%) or high (top 25%). Mural cell *FN1* expression was then compared between APOE genotypes (n = 8 per genotype) and between the low (n = 11, one outlier removed via Grubbs’ test) and high (n = 4) pathology groups. For each comparison, *FN1* expression was normalized to APOE3/3 or the low pathology group, respectively. Data points represent individuals. Bars are group means ± SEM. P-values were calculated using unpaired two-sample Student’s t-tests. The association between individual mural cell *FN1* expression and age at AD diagnosis (n = 16) was assessed via Pearson correlation analysis. **D.** miBrain experiments performed with additional isogenic pairs of iPSC-derived mural cells (iMCs) as previously described in Extended Figure 3B. ADRC scale bar, 25 µm. SAD scale bar, 50 µm. The area positive for fibronectin was normalized to nuclei and expressed relative to APOE3/3. Data points represent mean values from individual miBrains (n = 6 images per miBrain, n = 4–6 miBrains per genotype). **E.** Validation of *FN1* shRNA knockdown. qRT-PCR was performed on APOE4/4 iMCs in monoculture. Data points represent mean values from individual wells of iMCs normalized to scramble (3 qRT-PCR replicates per well, n = 3 wells per condition). Bars are mean group values ± SEM. P-values were calculated using unpaired two-sample Student’s t-tests. **F-I.** Additional outputs of NicheNet analysis from Figure 4A, B. **J.** Representative images of iMCs with and without TGFβ1 stimulation. Scale bar, 25 µm. For the control group, APOE3/3 and APOE4/4 iMCs were left untreated. For the experimental group, APOE3/3 iMCs were treated with 50 ng/mL TGFβ1 for 96 hours. iMCs were then fixed and stained for PDGFRβ and SMAD2. SMAD2 intensity inside the nuclear mask was quantified and expressed relative to APOE3/3 control. Data points represent mean values from individual wells of iMCs (6 images per well, 4 wells per condition). Bars are mean group values ± SEM. P-values were calculated using a one-way ANOVA with Dunnett’s test for multiple comparisons. **K.** Representative images of myofibroblast phenotypes in iMC monocultures after TGF-β inhibition. Scale bar, 50 µm. For the control group, APOE4/4 iMCs were left untreated. For the experimental group, APOE4/4 iMCs were treated with 10 µM SB431542 for 96 hours. iMCs were then fixed and stained for NG2, αSMA, and fibronectin. Intracellular αSMA and fibronectin areas were quantified as the positively stained regions within the NG2 mask, normalized to the total NG2 area, and expressed relative to APOE4/4 levels. Data points represent mean values from individual wells (n = 6 images per well, n = 4 wells per condition). Bars are mean group values ± SEM. P-values were calculated using unpaired two-sample Student’s t-tests.

## Supplementary Material

Supplement 1

Supplement 2

Supplement 3

Supplement 4

Supplement 5

Supplement 6

Supplement 7

Supplement 8

Supplement 9

Supplement 10

Supplement 11

Supplement 12

Supplement 13

Supplement 14

Supplement 15

Supplement 16

Supplement 17

## Figures and Tables

**Table:** Key Resources Table

REAGENT OR RESOURCE	SOURCE	IDENTIFIER
**Chemicals, peptides, and recombinant proteins**		
**Accutase**	Stemcell	Cat# 07920
**Activin A**	Peprotech	Cat# 120-14P
**Astrocyte Growth Supplement**	ScienCell	Cat#1852
**Astrocyte Medium (AM)**	ScienCell	Cat# 1801
**B27**	Gibco	Cat# 17504044
**B27 without Vitamin A**	Gibco	Cat# 12587010
**BDNF**	Peprotech	Cat# 450-02
**Biotin**	Sigma	Cat#B4639
**Blasticidin**	Gibco	Cat # A1113903
**BMP4**	Peprotech	Cat# 120-05ET
**CD Lipids**	Gibco	Cat# 11905031
**CHIR99021**	Tocris	Cat# 4423
**CNTF**	Peprotech	Cat# 450-13
**DAPT**	Cayman	Cat# 13197
**Dibutyryl cAMP**	Biogems	Cat# 1698950
**DMEM**	Gibco	Cat# 11965092
**DMEM/F12 with GlutaMAX**	Gibco	Cat# 10565018
**Doxycyline**	Millipore	Cat# D3072
**FGF-basic**	Peprotech	Cat# 100-18B
**Fibronectin**	R&D Systems	Cat# 4305-FNB-200
**Forkskolin**	R&D Systems	Cat# 1099/10
**Galunisertib**	Tocris	Cat# 6956
**GDNF**	Peprotech	Cat# 450-10
**Gelxtrex**	Gibco	Cat# A1413201
**GlutaMAX**	Gibco	Cat# 35050061
**HGF**	Peprotech	Cat# 100-39H
**Hoechst33342**	Thermo Scientific	Cat# 62249
**Human Endothelial SFM**	Gibco	Cat# 11111044
**IGF-1**	Peprotech	Cat# 100-11
**IL-34**	Peprotech	Cat# 200-34
**Insulin**	Sigma	Cat# I9278
**Laminin**	Gibco	Cat# 23017015
**L-Ascorbic Acid**	Fisher Scientific	Cat# BP351
**LDN193189**	Tocris	Cat# 6053
**Lipofectamine Stem Transfection Reagent**	Invitrogen	Cat# STEM00001
**Lycopersicon Esculentum (Tomato) Lectin (LEL, TL), DyLight 488**	Thermo Scientific	Cat# L32470
**MCSF**	Peprotech	Cat# 300-25
**MEM-Non-Essential Amino Acids**	Gibco	Cat#111400050
**N2**	Gibco	Cat# 17502048
**Neurobasal**	Gibco	Cat# 21103049
**NT3**	Peprotech	Cat# 450-03
**PDGF-AA**	Peprotech	Cat# 100-13A
**PDGF-BB**	Peprotech	Cat# AF-100-14B
**Penicillin-**	Gibco	Cat# 15140122
**Streptomycin**		
**Puromycin**	Gibco	Cat# A1113803
**Retinoic Acid**	MIllipore	Cat# R2625
**SAG**	Cayman	Cat# 11914
**SB431542**	Stemgent	Cat# 04-0010
**StemFlex**	Gibco	Cat# A3349401
**TGFB1**	Peprotech	Cat# 100-21
**TrypLE Select**	Gibco	Cat# 12563011
**VEGF-A**	Peprotech	Cat# 100-20
**Y-27632**	Tocris	Cat# 1254
**Antibodies**		
α**-smooth muscle actin**	R&D Systems	Cat# MAB1420
**Amyloid-**β **(12F4)**	BioLegend	Cat# 805501
**Amyloid-**β **(6E10)**	BioLegend	Cat# 803001
**Amyloid-**β **(D54D2)**	Cell Signaling Technology	Cat# 8243S
**CD31/PECAM-1**	R&D Systems	Cat# AF806
**CD31/PECAM-1**	R&D Systems	Cat# BBA7
**CD31/PECAM-1**	Thermo Scientific	Cat# MA5-29475
**Fibronectin**	R&D Systems	Cat# AF1918
**NG2**	Abcam	Cat# Ab255811
**PDGFRB**	Abcam	Cat# Ab69506
**pSmad2**	Millipore-Sigma	Cat# AB3849-I
**Smad2**	Cell Signaling Technology	Cat# 5339S
**VECAD/CD144**	R&D Systems	Cat# AF938
**Animals**		
B6.Cg-Apoeem2(APOE*)Adiuj/J (APOE*3 KI)	Jackson Labs	RRID: IMSR_JAX:029018
B6(SJL)-Apoetm1.1(APOE*4)Adiuj/J (APOE*4 KI)	Jackson Labs	RRID:IMSR_JAX:027894
**Cell lines**		
**AG09173 (MSSM3) iPSCs**	Massachusetts Institute of Technology	RRID: CVCL_4L66
**ADRC5 iPSCs**	UCI	
**sAD iPSCs**	Massachusetts Institute	
	of Technology	
**Plasmids**		
***FN1* shRNA plasmid for lentiviral production**	Thermo Scientific	TRCN0000293840
**piggyBac-rtTA (4th_Gen)-NGN2-2A-PURO-IRES-SNAP**	Addgene	Cat# 209077
**PB_iETV2_P2A_GFP_Puro**	Addgene	Cat# 168805
**Scramble shRNA plasmid for lentiviral production**	Millipore-Sigma	Cat# SHC016
**Software and algorithms**		
**CellProfiler (v4.2.8)**	Stirling et al.^126^	https://cellprofiler.org/
**CIBERSORTx**	Newman et al.^85^	https://cibersortx.stanford.edu/
**ClusterProfiler (v4.10.1)**	Yu et al.^127^	https://guangchuangyu.github.io/software/clusterProfiler/
**Condiments (v1.10.0)**	Roux de Bézieux et al.^128^	https://github.com/HectorRDB/condiments
**DAseq (v1.0.0)**	Zhao et al.^129^	https://github.com/KlugerLab/DAseq
**DoubletFinder (v2.0.4)**	McGinnis et al.^130^	https://github.com/chris-mcginnis-ucsf/DoubletFinder
**enrichR (v3.4)**	Chen et al.^77^	https://github.com/guokai8/EnrichR
**Fgsea (v1.28.0)**	Korotkevich et al.^131^	https://github.com/alserglab/fgsea
**Fiji (v2.3.0)**	Schindelin et al.^132^	imagej.net/software/fiji/
**GraphPad Prism (v10.4.1)**	GraphPad Software	graphpad.com
**GSVA (v1.50.5)**	Hänzelmann et al.^133^	https://github.com/rcastelo/GSVA
**Harmony (v1.2.3)**	Korsunsky et al.^134^	https://github.com/immunogenomics/harmony
**Limma (v3.58.1)**	Ritchie et al.^135^	https://bioconductor.org/packages/release/bioc/html/limma.html
**MAST (v1.28.0)**	Finak et al.^136^	https://github.com/RGLab/MAST
**Mgcv (v1.9.3)**	Wood^137^	https://cran.r-project.org/web/packages/mgcv/index.html
**miloR (v1.10.0)**	Dann et al.^58^	https://github.com/MarioniLab/miloR
**Msigdbr (v10.0.2)**	Dolgalev^138^	https://igordot.github.io/msigdbr/
**NicheNet (v2.1.5)**	Broweays et al.^93^	https://github.com/saeyslab/nichenetr
**Nikon NIS Elements**	Nikon	microscope.healthcare.nikon.com
**software**		
**R (v4.3.2)**	R Project	https://www.r-project.org/
**R Studio (v2024.04.2)**	Posit	https://posit.co/download/rstudio-desktop/
**Seurat (v5.2.1)**	Hao et al.^139^	https://satijalab.org/seurat/
**Slingshot (v2.10.0)**	Street et al.^140^	https://github.com/kstreet13/slingshot
**Other**		
**96-well plate with cover glass thickness polystyrene bottom**	Greiner Bio-One	655096
**Ti2E Ax R Confocal Microscope**	Nikon	microscope.healthcare.nikon.com
