# Supplementary material for: APOE4 promotes cerebrovascular fibrosis and amyloid deposition via a pericyte-to-myofibroblast transition
Source: bioRxiv. 2025 Sep 9:2025.09.04.674192. Preprint. [Version 1] doi: 10.1101/2025.09.04.674192 (PMC12439990; doi:10.1101/2025.09.04.674192)
Supplement: Supplement 1 [file media-1.docx]

**Supplementary Information Guide**

APOE4 promotes cerebrovascular fibrosis and amyloid deposition via a pericyte-to-myofibroblast transition

Braxton R. Schuldt^1,2,3^, Dominic Haworth-Staines^1,2,3^, Andrea Perez-Arevalo^1,2,3^, Diede W.M. Broekaart^1,2,4^, Leon Wang^1,2,3^, Georgia Gallagher^1,2,3^, Grace Rabinowitz^1,2,3^, Alison M. Goate^1,2,3^, Ana C. Pereira^1,2,4^, Joel W. Blanchard^1,2,3*^

1. Nash Family Department of Neuroscience and Friedman Brain Institute, Icahn School of Medicine at Mount Sinai, New York, NY, 10029, USA.

2. Ronald M. Loeb Center for Alzheimer’s Disease, Icahn School of Medicine at Mount Sinai, New York, NY, 10029, USA.

3. Black Family Stem Cell Institute, Icahn School of Medicine at Mount Sinai, New York, NY, 10029, USA

4. Department of Neurology, Icahn School of Medicine at Mount Sinai, New York, NY, 10029, USA

*To whom correspondence should be addressed: [joel.blanchard@mssm.edu](mailto:joel.blanchard@mssm.edu)

**Supplementary Table S1. Cluster markers of the major cerebrovascular cell types, related to Figure 1.**

| **Column** | **Explanation** |
| --- | --- |
| gene | Gene name |
| p_val | Nominal p-value |
| avg_log2FC | Log2(fold change) in gene expression in the cluster of interest vs. the other clusters |
| pct.1 | Percent of cells in the cluster of interest expressing the gene |
| pct.2 | Percent of cells outside the cluster of interest expressing the gene |
| p_val_adj | Adjusted p-value |
| cluster | Cluster of interest |

**Supplementary Table S2. Significant differentially abundant cell neighborhoods in APOE4 carriers vs. non-carriers for the major cerebrovascular cell types via miloR analysis, related to Figure 1.**

| **Column** | **Explanation** |
| --- | --- |
| Nhood | The number assigned to that neighborhood in miloR |
| logFC | The log fold change in abundance for that neighborhood in APOE4 carriers vs. non-carriers (logFC > 0 indicates enrichment in APOE4 carriers) |
| logCPM | Average log normalized cell counts across all individuals |
| F | The f-statistic from the quali-likelihood F-test |
| PValue | Nominal p-value |
| FDR | P-value adjusted with Benjamini & Hochberg method |
| SpatialFDR | The FDR adjusted for spatial graph overlapds between neighborhoods |
| celltype | The cell type in which the neighborhood is assigned to |
| celltype_fraction | The proportion of cells in that neighborhood that belong to the assigned cell type |

**Supplementary Table S3. Cluster markers of the mural cell subtypes, related to Figure 1.**

| **Column** | **Explanation** |
| --- | --- |
| gene | Gene name |
| p_val | Nominal p-value |
| avg_log2FC | Log2(fold change) in gene expression in the cluster of interest vs. the other clusters |
| pct.1 | Percent of cells in the cluster of interest expressing the gene |
| pct.2 | Percent of cells outside the cluster of interest expressing the gene |
| p_val_adj | Adjusted p-value |
| cluster | Cluster of interest |

**Supplementary Table S4. Differential gene expression analysis in mural cell subclusters of APOE4 carriers vs. non-carriers via MAST, related to Figure 1.**

| **Column** | **Explanation** |
| --- | --- |
| gene | Gene name |
| p_val | Nominal p-value |
| avg_log2FC | Log2(fold change) in gene expression in APOE4 carriers vs. non-carriers for the given cluster (avg_log2FC > 0 indicates upregulation in APOE4 carriers) |
| pct.1 | Percent of APOE4 carrier cells in the given cluster expressing the gene |
| pct.2 | Percent of APOE3/3 cells in the given cluster expressing the gene |
| p_val_adj | Adjusted p-value |
| cluster | Cluster of interest |

**Supplementary Table S5. Gene set enrichment analysis of APOE4 carrier vs. non-carrier MAST results from S4, related to Figure 1.**

| **Column** | **Explanation** |
| --- | --- |
| pathway | Name of pathway |
| pval | Nominal p-value |
| padj | Adjusted p-value |
| ES | Enrichment score |
| NES | Normalized enrichment score in APOE4 carriers vs. non-carriers (NES > 0 indicates enrichment in APOE4 carriers) |
| size | Size of the pathway (number of genes in that pathway) |
| leadingEdge | Genes that are driving the enrichment |

**Supplementary Table S6. Pseudobulked differential gene expression analysis in SMC_2 cluster of APOE4 carriers vs. non-carriers via limma, related to Figure 1.**

| **Column** | **Explanation** |
| --- | --- |
| gene | Gene name |
| logFC | Log2(fold change) in gene expression in APOE4 carriers vs. non-carriers (logFC > 0 indicates upregulation in APOE4 carriers) |
| AveExpr | Log2 expression level across all individuals |
| t | Moderated t-statistic |
| P.Value | Nominal p-value |
| adj.P.Val | Adjusted p-value |
| B | Log-odds that the gene is differentially expressed |

**Supplementary Table S7. Gene set enrichment analysis of APOE4 carrier vs. non-carrier SMC_2 limma results from S6, related to Figure 1.**

| **Column** | **Explanation** |
| --- | --- |
| pathway | Name of pathway |
| pval | Nominal p-value |
| padj | Adjusted p-value |
| ES | Enrichment score |
| NES | Normalized enrichment score in APOE4 carriers vs. non-carriers (NES > 0 indicates enrichment in APOE4 carriers) |
| size | Size of the pathway (number of genes in that pathway) |
| leadingEdge | Genes that are driving the enrichment |

**Supplementary Table S8. Cell type annotation of APOE4 SMC_2 gene signature via enrichR, related to Figure 1.**

| **Column** | **Explanation** |
| --- | --- |
| Term | Name of cell type |
| Overlap | Fraction of genes overlapping between the APOE4 gene list and the cell type |
| P.value | Nominal p-value |
| Adjusted.P.value | Adjusted p-value |
| Odds.Ratio | Association between APOE4 gene list and the cell type gene list |
| Combined.Score | Natural log of p-value multiplied by the z-score (deviation from expected rank) |
| Genes | Overlapping genes between the APOE4 gene list and the cell type gene list |

**Supplementary Table S9. Differential gene expression analysis in APOE4-enriched DAseq subregion vs. remaining SMC_2 cells via MAST, related to Figure 1.**

| **Column** | **Explanation** |
| --- | --- |
| gene | Gene name |
| p_val | Nominal p-value |
| avg_log2FC | Log2(fold change) in gene expression in APOE4-enriched DAseq subregion vs. remaining SMC_2 cells (avg_log2FC > 0 indicates upregulation in APOE4 region) |
| pct.1 | Percent of cells in APOE4 region expressing the gene |
| pct.2 | Percent of remaining SMC_2 cells expressing the gene |
| p_val_adj | Adjusted p-value |

**Supplementary Table S10. ClusterProfiler pathway enrichment analysis of differentially expressed genes in APOE4-enriched DAseq subregion vs. remaining SMC_2 cells from S9, related to Figure 1.**

| **Column** | **Explanation** |
| --- | --- |
| ONTOLOGY | Ontology aspect in which that term belongs (cellular compartment [CC], molecular function [MF], or biological process [BP]) |
| ID | Ontology term ID |
| Description | Description of the ontology term |
| Gene ratio | Ratio of provided genes overlapping with the genes in the GO term |
| BgRatio | Ratio of all genes overlapping with the genes in the GO term |
| pvalue | Nominal p-value |
| p.adjust | Adjusted p-value |
| geneID | Overlapping genes between provided gene list and genes in the ontology term |
| enriched_celltype | Region in which the ontology term is enriched (Myofibroblast = APOE4-enriched DAseq subregion [genes with avg_log2FC > 0 in S9]; SMC_2 = rest of SMC_2 cells [genes with avg_log2FC < 0 in S9)] |

**Supplementary Table S11. Cell type annotation of APOE4-enriched DAseq subregion via enrichR, related to Figure 1.**

| **Column** | **Explanation** |
| --- | --- |
| Term | Name of cell type |
| Overlap | Fraction of genes overlapping between the APOE4 gene list and the cell type |
| P.value | Nominal p-value |
| Adjusted.P.value | Adjusted p-value |
| Odds.Ratio | Association between APOE4 gene list and the cell type gene list |
| Combined.Score | Natural log of p-value multiplied by the z-score (deviation from expected rank) |
| Genes | Overlapping genes between the APOE4 gene list and the cell type gene list |

**Supplementary Table S12. Differential gene expression analysis of APOE4/4 iPSC-derived mural cells (iMCs) vs. APOE3/3 iMCs via limma, related to Figure 2.**

| **Column** | **Explanation** |
| --- | --- |
| gene | Gene name |
| logFC | Log2(fold change) in gene expression in APOE4/4 iMCs vs. APOE3/3 iMCs (logFC > 0 indicates upregulation in APOE4/4 iMCs) |
| AveExpr | Log2 expression level across all samples |
| t | Moderated t-statistic |
| P.Value | Nominal p-value |
| adj.P.Val | Adjusted p-value |
| B | Log-odds that the gene is differentially expressed |

**Supplementary Table S13. ClusterProfiler pathway enrichment analysis of differentially expressed genes in APOE4/4 iMCs vs. APOE3/3 iMCs from S12, related to Figure 2.**

| **Column** | **Explanation** |
| --- | --- |
| ONTOLOGY | Ontology aspect in which that term belongs (cellular compartment [CC], molecular function [MF], or biological process [BP]) |
| ID | Ontology term ID |
| Description | Description of the ontology term |
| Gene ratio | Ratio of provided genes overlapping with the genes in the GO term |
| BgRatio | Ratio of all genes overlapping with the genes in the GO term |
| pvalue | Nominal p-value |
| p.adjust | Adjusted p-value |
| geneID | Overlapping genes between provided gene list and genes in the ontology term |
| direction | Whether the ontology term is upregulated or downregulated in APOE4/4 iMCs (Upregulated = genes with logFC > 0 in S12; Downregulated = genes with logFC < 0 in S12) |

**Supplementary Table S14. Differential gene expression analysis in fibroblasts of APOE4 carriers vs. non-carriers via MAST, related to Figure 3.**

| **Column** | **Explanation** |
| --- | --- |
| gene | Gene name |
| p_val | Nominal p-value |
| avg_log2FC | Log2(fold change) in gene expression in APOE4 carriers vs. non-carriers (avg_log2FC > 0 indicates upregulation in APOE4 carriers) |
| pct.1 | Percent of APOE4 carrier fibroblast cells expressing the gene |
| pct.2 | Percent of APOE3/3 fibroblast cells expressing the gene |
| p_val_adj | Adjusted p-value |

**Supplementary Table S15. Gene set enrichment analysis of APOE4 carrier vs. non-carrier fibroblast MAST results from S14, related to Figure 3.**

| **Column** | **Explanation** |
| --- | --- |
| pathway | Name of pathway |
| pval | Nominal p-value |
| padj | Adjusted p-value |
| ES | Enrichment score |
| NES | Normalized enrichment score in APOE4 carriers vs. non-carriers (NES > 0 indicates enrichment in APOE4 carriers) |
| size | Size of the pathway (number of genes in that pathway) |
| leadingEdge | Genes that are driving the enrichment |

**Supplementary Table S16. NicheNet ligand activities in APOE4 myofibroblasts, related to Figure 4.**

| **Column** | **Explanation** |
| --- | --- |
| test_ligand | Ligand name |
| aupr | Area under the precision-recall curve (AUPR) |
| aupr_corrected | Corrected AUPR value |
